# Fruiting Body Formation in *Volvariella volvacea* Can Occur Independently of Its *MAT-A*-Controlled Bipolar Mating System, Enabling Homothallic and Heterothallic Life Cycles

**DOI:** 10.1534/g3.116.030700

**Published:** 2016-05-16

**Authors:** Bingzhi Chen, Arend F. van Peer, Junjie Yan, Xiao Li, Bin Xie, Juan Miao, Qianhui Huang, Lei Zhang, Wei Wang, Junsheng Fu, Xiang Zhang, Xiaoyin Zhang, Fengli Hu, Qingfang Kong, Xianyun Sun, Feng Zou, Hanxing Zhang, Shaojie Li, Baogui Xie

**Affiliations:** * Mycological Research Center, Fujian Agriculture and Forestry University, Fuzhou 350002, China; † Institute of Microbiology, State Key Laboratory of Mycology, Chinese Academy of Sciences, Beijing 100080, China

**Keywords:** *Volvariella volvacea*, genomic analysis, mating-type system, aneuploidy, amphithallic

## Abstract

*Volvariella volvacea* is an important crop in Southeast Asia, but erratic fruiting presents a serious challenge for its production and breeding. Efforts to explain inconsistent fruiting have been complicated by the multinucleate nature, typical lack of clamp connections, and an incompletely identified sexual reproductive system. In this study, we addressed the life cycle of *V. volvacea* using whole genome sequencing, cloning of *MAT* loci, karyotyping of spores, and fruiting assays. Microscopy analysis of spores had previously indicated the possible coexistence of heterothallic and homothallic life cycles. Our analysis of the *MAT* loci showed that only *MAT-A*, and not *MAT-B*, controlled heterokaryotization. Thus, the heterothallic life cycle was bipolar. Karyotyping of single spore isolates (SSIs) using molecular markers supported the existence of heterokaryotic spores. However, most SSIs were clearly not heterokaryotic, yet contained structural variation (SV) markers relating to both alleles of both parents. Heterokaryons from crossed, self-sterile homokaryons could produce fruiting bodies, agreeing with bipolar heterothallism. Meanwhile, some SSIs with two different *MAT-A* loci also produced fruiting bodies, which supported secondary homothallism. Next, SSIs that clearly contained only one *MAT-A* locus (homothallism) were also able to fruit, demonstrating that self-fertile SSIs were not, per definition, secondary homothallic, and that a third life cycle or genetic mechanism must exist. Finally, recombination between SV markers was normal, yet 10 out of 24 SV markers showed 1:2 or 1:3 distributions in the spores, and large numbers of SSIs contained doubled SV markers. This indicated selfish genes, and possibly partial aneuploidy.

Sexual reproduction in fungi can be classified into homothallism (inbreeding/self-fertile) and heterothallism (outbreeding/self-sterile). This is based on whether or not a compatible mating-type (*MAT*) locus from a mating partner is required to instigate and complete the sexual cycle. Fungi within the basidiomycetes have developed unique mating-type systems consisting of two unlinked, functionally different, sex-determining *MAT* loci. These loci can be recombined into four different mating-types during meiosis; the tetrapolar mating-type system (for recent reviews, see Fraser *et al.* 2007; Hsueh and Heitman 2008). In mushroom-forming fungi (Agaricomycetes), complexity of the mating-type system has evolved even further, with the possibility of multiple subloci for both *MAT-A* and *MAT-B*, and each (sub) locus potentially carrying manifold alleles, *i.e.*, multiallelic subloci (Whitehouse 1949; Raper 1966). Typically, the *MAT-A* locus of homobasidiomycetes contains a set of divergently transcribed homeodomain (HD) protein encoding genes, one HD1 type and one HD2 type (Raper 1968; Brown and Casselton 2001). The *MAT-A* locus governs genes of the *A* mating-type pathway, which control initiation of clamp cell formation and synchronized nuclear division (Raper 1968; Kües *et al.* 1994; Casselton and Olesnicky 1998; Brown and Casselton 2001). Genes at the *MAT-B* locus encode sets of pheromone receptor genes with one or more associated pheromone precursors that regulate clamp cell fusion and nuclear migration (Casselton and Olesnicky 1998; Raudaskoski and Kothe 2010).

Notwithstanding a tetrapolar origin (Burnett 1975, for discussion see Fraser *et al.* 2004, 2007; Hsueh and Heitman 2008), mushrooms with tetrapolar (∼65%), bipolar (∼25%), as well as homothallic (∼10%) mating systems have been found distributed throughout the homobasidiomycete phylogeny (Raper 1966; Hibbett and Donoghue 2001; James *et al.* 2006). In several higher basidiomycetes, bipolarity has been traced to loss of mating specificity of the *MAT-B* locus. Whereas *MAT-B* is complete and present (and probably ’active’), only the *MAT-A* locus segregates in a mating-type specific manner (Aimi *et al.* 2005; James *et al.* 2006, 2011).

Development of homothallism, or the ability to self-mate, is not clearly understood (Lin and Heitman 2007). Monokaryotic fruiting of otherwise heterothallic species is regularly observed in colonies of mushroom forming fungi that are exposed to prolonged stress or treatment with certain chemicals, or due to mutations in the mating type pathways (*e.g.*, Esser and Stahl 1973; Leslie and Leonard 1980; Gibbins and Lu 1984). Other examples of homothallism in mushroom forming fungi comprise pseudohomothallism, in which two instead of four basidiospores emerge on the basidia, and each spore contains two compatible postmeiotic nuclei (Kerrigan *et al.* 1993; Callac *et al.* 1996) and exhibits mating-type switching (Labarere and Noel 1992). Species harboring a combination of different mating systems are also known (*e.g.*, *Agaricus* species), and are referred to as amphithallic (Labarere and Noel 1992; Kerrigan *et al.* 1993; Callac *et al.* 1996; Lin and Heitman 2007; Hsueh and Heitman 2008).

*Volvariella volvacea* (Bull, ex, Fr.) Sing., better known as Chinese mushroom, or the Straw mushroom, is an edible mushroom that ranks sixth in worldwide mushroom production (Chang 1999; Zhang 2009) and has important dietary benefits and pharmaceutical applications (Chang 1978; Kishida *et al.* 1992; Hsu *et al.* 1997; She *et al.* 1998). The unclear mating-type system of this mushroom, as well as its irregular fruiting behavior, represents a considerable problem for production and strain improvement. At the same time, our incapability to classify *V. volvacea* according to known mating-type systems suggests that our understanding of these mechanisms is incomplete. *V. volvacea* grows through means of multinucleate hyphae (Li 1982). Clamp connections are absent in homo- and heterokaryons as well as in any other examined mushroom tissue (Chang and Yau 1971; Li 1982; Chiu 1993; Chiu and Moore 1999), and nuclei are irregularly shaped and exhibit asynchronous nuclear phases within a single hyphal compartment (Chiu 1993; Chiu and Moore 1999). The basidia show typical meiotic figures both in fruiting bodies formed from (presumed) homokaryons and from heterokaryons (Chang and Ling 1970; Wells 1977; Chiu 1993). They are generally tetrasporic, although additional mono-, di-, tri-, and pentasporic basidia have also been reported (Chang and Yau 1971; Li 1982). Basidia were reported to be anucleate after spore discharge, and basidiospores are predominantly mononuclear (>85%) (Chang and Yau 1971; Li 1982; Li and Chang 1991; Chiu 1993). Binuclear spores are also observed, but it is unclear if these are entirely heterothallic, or result partially from postmeiotic nuclear divisions within spores. Differing reports describe self-fertility of homokaryotic mycelia obtained from single spores in frequencies that are much higher than estimated percentages of heterokaryotic spores (Chang and Yau 1971; Li and Chang 1979; Chang *et al.* 1981). Moreover, crossing experiments with varying outcomes (Chang and Yau 1971; Chang 1978; Royse *et al.* 1987; Bao *et al.* 2013) have indicated the existence in *V. volvacea* of homothallism as well as heterothallism, the latter being later confirmed by segregation studies, electrophoretic karyotyping (Chiu and Moore 1999), and microspectrophotometric observations (Chiu 1993). The analysis of the genome sequence of *V. volvacea* V23-1 (Bao *et al.* 2013) revealed mating type specific *MAT-A*, and conserved, incomplete *MAT-B* loci. Based on *MAT-A*-specific karyotyping, it was further concluded that *V. volvacea* was pseudo or secondary homothallic. Yet, this did not explain the contradicting numbers of fertile single spore isolates (SSIs) and predicted heterokaryotic spores.

In this study, we addressed the life cycle of *V. volvacea* using whole genome sequencing, cloning of *MAT* loci, and karyotyping of spores with genetic markers. We determined *MAT* locus polymorphism in a series of strains, performed crosses establishing the role of the respective *MAT* loci in mating, and determined the presence of three coexisting, homo- and heterothallic life cycles.

## Materials and Methods

### V. volvacea strains

Strains used in this study are listed in Table 1. Strains PY1, V0049, and V0238 are common commercial cultivars of Fujian Province, China. Homokaryotic strains PYd15 and PYd21, the genomes of which have been sequenced, were derived from basidiospores of PY1 (Chen *et al.* 2013). Heterokaryotic strain H1521 resulted from a new cross between homokaryons PYd15 and PYd21. Colony morphology, fruiting tests, and molecular markers confirmed that H1521 was the heterokaryotic product of strains PYd15 and PYd21.

**Table 1:** *Volvariella volvacea* strains, corresponding homokaryons, and identified *MAT-A* loci

Strain	Origin	Homokaryons	*Mat A*
PY1	Fujian province, commercial	PYd15	*A1*
		PYd21	*A2*
HNL	Fujian province, wild	HNL-1	*A2*
		HNL-3	*A3*
V0032	Fujian province, wild	V0032-3	*A6*
		V0032-6	*A4*
V0076	Fujian province, wild	V0076-1	*A6*
		V0076-7	*A7*
V0077	Sichuan province, wild	V0077-1	*A5*
		V0077-16	*A2*
V0124	Sichuan province, wild	V0124-1	*A2*
		V0124-7	*A8*
V0049	Fujian province, commercial	V0049-4	*A7*
		V0049-6	*A6*
V0238	Fujian province, commercial	V0238-8	*A6*
		V0238-9	*A7*
V23	Fujian province, commercial	V23-1	*A7*

Homokaryotic, SSIs were obtained from seven additional heterokaryotic strains (indicated in Table 1), including five wild isolates from two different Chinese provinces, and two commercial strains. Strains were deposited at the Fujian Edible Fungi Germplasm Resource Collection Center of China under the indicated accession numbers (Table 1).

### Media and cultivation conditions

*V. volvacea* strains were grown routinely at 32° on potato dextrose agar (PDA; 200 g/l boiled and sieved peeled potato, 20 g/l glucose, 20 g/l agar). Mycelium for genomic DNA and RNA extractions was cultivated in liquid potato dextrose broth (PDB; equal to PDA without agar) at 32°, shaking at 150 rpm, for 4 or 7 d. For fruiting assays, strains were cultivated on rice straw compost according to Chen *et al.* (2004). Beds were observed for 30 d after inoculation to determine if strains produced fruiting bodies. Primordia of H1521 for RNA extraction were harvested at d 8 after inoculation, flash frozen in liquid nitrogen and stored at –80°. For mating tests, strains were inoculated 2 cm apart on PDA. Interaction zones were excised and transferred to new plates, and single hyphae of 1/3 plate-size colonies were selected under a microscope for subsequent cultivation and DNA extraction. Single spore colonies were obtained by selecting single hyphae from germinating spores under a microscope, followed by separate cultivation.

### Genome sequencing and annotation of A and B mating-type genes

Genomic DNA of *V. volvacea* strain PYd15 was extracted using a modified CTAB method (Stajich *et al.* 2010), sequenced (BGI-Shenzhen, http://www.genomics.cn), and assembled against the reference genome sequence of PYd21 (Chen *et al.* 2013). Briefly, a paired-end library of DNA fragments with 505-bp insert size and read lengths of 90 bp was generated from strain PYd15 and aligned to the reference genome. Genes were predicted using Eukaryotic GeneMark-ES version 2.3 (Ter-Hovhannisyan *et al.* 2008) and Augustus 2.5.5 (Stanke *et al.* 2006). Predicted gene models were functionally annotated based on homology with nonredundant genes from NCBI.

*MAT-A* genes of homokaryon PYd15 and PYd21 were identified based on homology with *MAT-A* genes of *V. volvacea* V23-1 (GenBank accessions: HD1, AEO99207.1; HD2, AIN76768.1, Bao *et al.* 2013). *MAT-B* genes (unpublished for *V. volvacea*) were identified based on homology with Rcb1.3, AAO17255; rcb3.6, CAA71962; rcb2.6, CAA71963; rcb1.6, CCA71964; Rcb2.43, AAQ96345; Rcb2.44, AAQ96344; Rcb3.42, AAF01420; and Rcb2.42, AAF01419 (*Coprinopsis cinerea* okayama7#130). Predicted mating-type gene models were confirmed through transcriptome reads as described previously (Chen *et al.* 2013). Pheromone genes were predicted within the 5-kb flanking sequences of pheromone receptor genes. NCBI’s ORF finder (http://www.ncbi.nlm.nih.gov/gorf/gorf.html) and InterPro (Quevillon *et al.* 2005) were used to predict ORFs [20–100 amino acids (AA)], which were screened for C- terminal “-CAAX” motifs (C, cysteine; A, aliphatic residue; X, any AA), and “ER or ED” motifs (∼10–12 AA) upstream of the “-CAAX” motif (Olesnicky *et al.* 1999).

*MAT-A* and *MAT-B* loci of monokaryotic progeny from seven additional heterokaryotic *V. volvacea* strains (Table 1) were amplified by long-distance PCR (Barnes 1994) using LA *Taq* (Takara) and primers LP-f/r (Supplemental Material, Table S1A and File S1), designed on conserved *MAT-A* locus flanking sequences of PYd21 scaffold 124 (LP-f, 1180282-1180303; LP-r 1187208-1187187, total scaffold 1425 kb, amplified length of 6926 bp) essentially following James *et al.* (2004). Similarly, *MAT-B* loci were amplified using primer pairs VvSTE3.1-f/r, VvSTE3.2-f/r, VvSTE3.3-f/r and VvSTE3.4-f/r (Table S1A). PCR products were gel-purified with a TIANGEN Universal DNA Purification Kit, and sequenced (Sangon Biotech, China). Gene models were identified and annotated as described above. Transmembrane domains of pheromone receptors were predicted using TMHMM Server version 2.0 (http://www.cbs.dtu.dk/services/TMHMM-2.0/) (Center for Biological Sequence Analysis, Technical University of Denmark, Lyngby, Denmark).

Coiled-coil dimerization motifs (CCDs) of HD proteins were identified using COILS (window size 14, Lupas *et al.* 1991), nuclear localization sequences (NLSs) using PSORT II (Nakai and Kanehisa 1992, http://psort.hgc.jp/form2.html), and HDs using InterPro (Quevillon *et al.* 2005). Alignment of DNA sequences was performed with DNAMAN (Huang and Zhang 2004).

### PCR and quantitative PCR analysis

Mating of homokaryons was confirmed by PCR on genomic DNA of crossed strains (see above) using *MAT-A*-specific markers (Table S1B). Sequence-characterized amplified regions (SCAR) and structural variation (SV) markers for analysis of single spore isolates were described by Wang *et al.* (2015).

For qRT-PCR, RNA was extracted using the E.Z.N.A Plant RNA Kit (Omega, Biotech, Norcross, Ga) according to the manufacturer’s protocol. Extracted RNA was quantified using an ND 1000 Spectrophotometer (NanoDrop Technologies, Wilmington, DE). Only RNA samples with A260/A280 ratios between 1.9 and 2.1, and A260/A230 ratios greater than 2.0 were used for further analysis. cDNA was synthesized using the TransScript All-in One First-Strand cDNA Synthesis SuperMix for qRT-PCR (One-Step gDNA Removal) Kit (TransGen Biotech, China), according to the manufacturer’s protocol. Primers for qRT-PCR were designed across introns using Primer Premier Version 5.0 (Table S1C). qRT-PCR was performed using a CFX96 Real-Time PCR Detection System (Bio-Rad, CA), with SsoAdvanced SYBR Green Supermix (Bio-Rad). Reactions followed denaturation for 10 sec at 95°, 40 cycles of 5 sec at 95°, and 30 sec at primer-specific annealing temperatures. The glyceraldehyde-3-phosphate dehydrogenase gene (*gapdh*) was used as an internal control gene. qRT-PCR data analysis was performed with the 2^–△△Ct^ method (Livak and Schmittgen 2001).

### Phylogenetic analysis of pheromone receptor genes

Phylogenetic relations of pheromone receptors were analyzed using sequences from *V. volvacea*, *Laccaria bicolor*, *S*chizophyllum *commune*, *C. cinerea* and *Flammulina velutipes*. Protein sequences were aligned (MUSCLE, Edgar 2004), ambiguously aligned regions were removed, and the remaining ∼180-AA regions were assembled in a maximum-likelihood three with MOLPHY (Dereeper *et al.* 2008).

### Data availability

The data from this study were deposited with NCBI GenBank under accession numbers JN578700.1, JN578701.1, JX982139.1, JX982140.1, JX982141.1, JX982142.1, KX022590, KX022591, KX022592, KX022593, KX022594, KX022595, KX022596, KX022597, KX022598, KX022599, KX022600, and KX022601.

## Results

### Identification of MAT-A genes in V. volvacea genome sequences

Two genomes of mating compatible strains PYd21 (Chen *et al.* 2013) and PYd15 (this study) were screened for *MAT-A* genes, and compared to the genome of strain V23-1 (Bao *et al.* 2013). The PYd15 genome draft was assembled using the genome of strain PYd21 as a reference. Clean reads, 1200 Mb in length, of a 90 bp paired-end (505 bp average insert size) library generated 1923 scaffolds (N50 = 91 kb, contigs < 200 bp were excluded) that contained 9087 predicted genes (∼30 × coverage, 1200 Mb/38 Mb). Coverage and scaffold size of the PYd15 genome draft were lower than that of *V. volvacea* PYd21 (37.2 Mb, 302 scaffolds, N50 499 kb, 11,534 predicted genes, 90 × coverage), and V23-1 (35.7 Mb, 62 scaffolds, N50 388 kb, 11,084 predicted genes), causing a lower number of predicted genes. The presence of homologs for all known *V. volvacea* mating type genes in strain PYd15 indicated sufficient coverage for mating type analysis.

Homology searches with known HD1 and HD2 proteins of *V. volvacea* V23-1 (gene *vv-HD1*^-V23-1^ and gene *vv-HD2*^-V23-1^) revealed one *MAT-A* locus with a single pair of divergently transcribed HD1 and HD2 encoding genes in the genome of PYd15 and PYd21 [scaffold 96 (359 kb), bps 212079–213803 and scaffold 124 (142.5 kb), bps 1184735–1186284, respectively]. Cross comparisons using the newly identified HD proteins revealed no other *MAT-A* genes in PYd15, PYd21, and V23-1. Gene models (intron/exon boundaries) of the *VvHd* genes were confirmed using transcriptomics data (Chen *et al.* 2013).

The predicted HD1 proteins of PYd15 and PYd21 were 501 AA (1605 bp) and 505 AA (1620 bp) long (Figure S1 and Figure S2). HD2 proteins were slightly shorter in both strains (Figure S3 and Figure S4), counting 442 AA (1485 bp) and 456 AA (1420 bp). These sizes were comparable to the reported HD1 and HD2 proteins in V23-1 (Figure S1, Figure S2, Figure S3, and Figure S4; Bao *et al.* 2013). New HD protein encoding genes were named *vv-HD1*^–PYd15^, *vv-HD1*^–PYd2^*^1^*, *vv-HD2*^–PYd15^, and *vv-HD2*^–PYd21^ (GenBank accessions: JN578700.1 and JN578701.1), the originating strains indicated in superscript.

### Comparison of A mating-type regions in V. volvacea genomes

**Figure 1:**
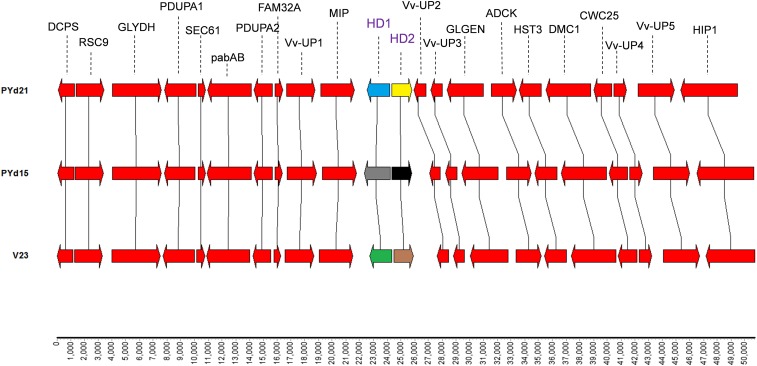
CHROMOMAPPER (Niculita-Hirzel and Hirzel 2008) comparison of genomic regions containing the *MAT-A* locus from *V. volvacea* strains PYd21, PYd15, and V23-1, showing the *MAT-A* locus (HD genes in colored arrows, and purple text), and conserved *MAT-A* locus flanking genes including the *mitochondrial intermediate peptidase* gene (MIP), the Sec61 complex subunit gene (SEC61), the *glycine dehydrogenase* gene (GLYDH), and the glycogenin-2β gene (GLGEN). Black lines connect homologous genes (depicted as red arrows). Relative gene positions (scale in bp) are indicated at the bottom.

Alignment of 50 kb genomic regions that surrounded the *MAT-A* loci in PYd15, PYd21, and V23-1 revealed very high sequence conservation (PYd15/PYd21, 90.74%; PYd15/V23-1, 90.16%; PYd21/V23-1, 90.21%). Synteny mapping showed that the only genes differing in the three 50 kb regions were the respective alleles of the *vv-Hd* genes (Figure 1). The high conservation of these regions over different strains with different mating types indicated that the organization presented is common for *V. volvacea*, which was supported by compatibility of conserved *MAT-A* primers with all tested additional strains (described below). Therefore, the observed differences with *MAT-A* regions of other mushrooms as reported in Bao *et al.* (2013) are expected to be characteristic for *V. volvacea*. High sequence polymorphism of HD1 and HD2 protein-encoding genes, and their mating type specific combinations, indicated that *MAT-A* was involved in mating type discrimination.

### Identification of MAT-A genes in additional V. volvacea strains

*MAT-A* loci from additional heterokaryotic *V. volvacea* strains were cloned using long distance PCR with primers that matched conserved sequences in the *mip* gene (primer LP-f) and *Vvup2* gene (primer LP-r), (Figure 1, Table S1A, and Figure S5). SSIs of seven heterokaryotic strains (HNL, V0032, V0076, V0077, V0124, V0049, and V0238, Table 1) were selected for amplification of *MAT-A* loci, followed by restriction enzyme fragment length polymorphism (RFLP) analysis, which distinguished a total of seven different size-based groups (Figure S6). Each of the different size groups was sequenced and annotated (sequences shown in Figure S1 and Figure S2), resulting in the identification of five new *MAT-A* subtypes. Two of the seven cloned *MAT-A* loci were identical to PYd21 (homokaryons HNL-1, V0077-16, and V0124-1), and strain V23-1 (V0076-7, V0049, and V0238-9). The *MAT-A l*ocus of PYd15 was not found among the newly cloned subtypes, bringing the total of identified *V. volvacea MAT-A* loci to eight (three genomic and five subcloned loci; GenBank accessions: KX022590, KX022591, KX022592, KX022593, KX022594, KX022595, KX022596, KX022597, KX022598, KX022599, KX022600, and KX022601).

### Analysis of MAT-A genes and proteins

**Figure 2:**
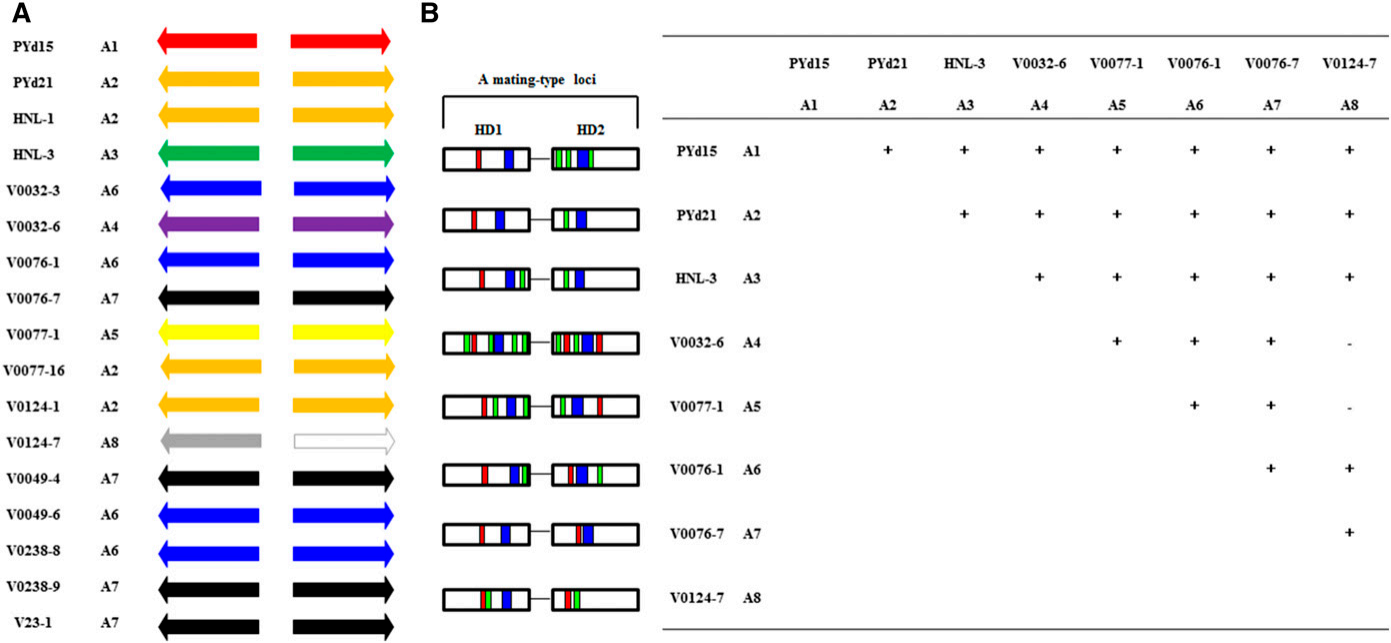
Overview of the *MAT-A* loci in single spore isolates from *V. volvacea*. (A) Different *MAT-A* loci (A1–A8) are indicated by differently colored arrows. Strain names equal strain collection registration codes. (B) Overview of the HD1 and HD2 protein structures of the eight different mating types, and their compatibility in mating assays. Strain names are indicated above and left of the cross scheme, together with their corresponding mating types. Ability to form a heterokaryon (as determined by *MAT-A* gene specific PCRs) is indicated with “+”, while inability to form a heterokaryon is indicated by “−,” (*e.g.*, A4 and A5 with A8). Structure of the *MAT-A* loci is given with arrows for HD1 and HD2 proteins, indicating their relative direction, and red squares for NLSs, blue squares for HDs and green squares for Di domains.

*Vv-Hd1* and *vv-Hd2* alleles constituted unique, fixed couples for all eight different *MAT-A* loci. The alignments of the gene and protein sequences clearly showed high DNA and AA polymorphism, an important indicator that the *MAT-A* genes were involved in mating in *V. volvacea*. Prediction of CCDs, dimerization motifs (Di), and NLSs revealed considerable differences between the various HD proteins (Figure 2, Figure S2, and Figure S4). All HD1 proteins contained a single predicted NLS, as well as three of the HD2 proteins (the HD2 of mating type A4 contained two predicted NLSs). HD2 proteins of mating types A1, A2, and A3 contained no predicted NLS, suggesting that the NLS might be more important in HD1 proteins. Predicted Di motifs varied greatly among the different HD proteins (from zero to four), and no Di motifs were found in either HD1 or HD2 of mating type A7. However, this mating type was clearly still functional (Figure 2). Di predictions were therefore regarded as merely indicative. Surprisingly, the HD2 domain of mating type A8 (strain V0124-7, HD2^–V0124-7^) was found to be incomplete, and therefore expected to be no longer able to properly bind DNA.

### Identification of MAT-B genes in V. volvacea genome sequences

**Figure 3:**
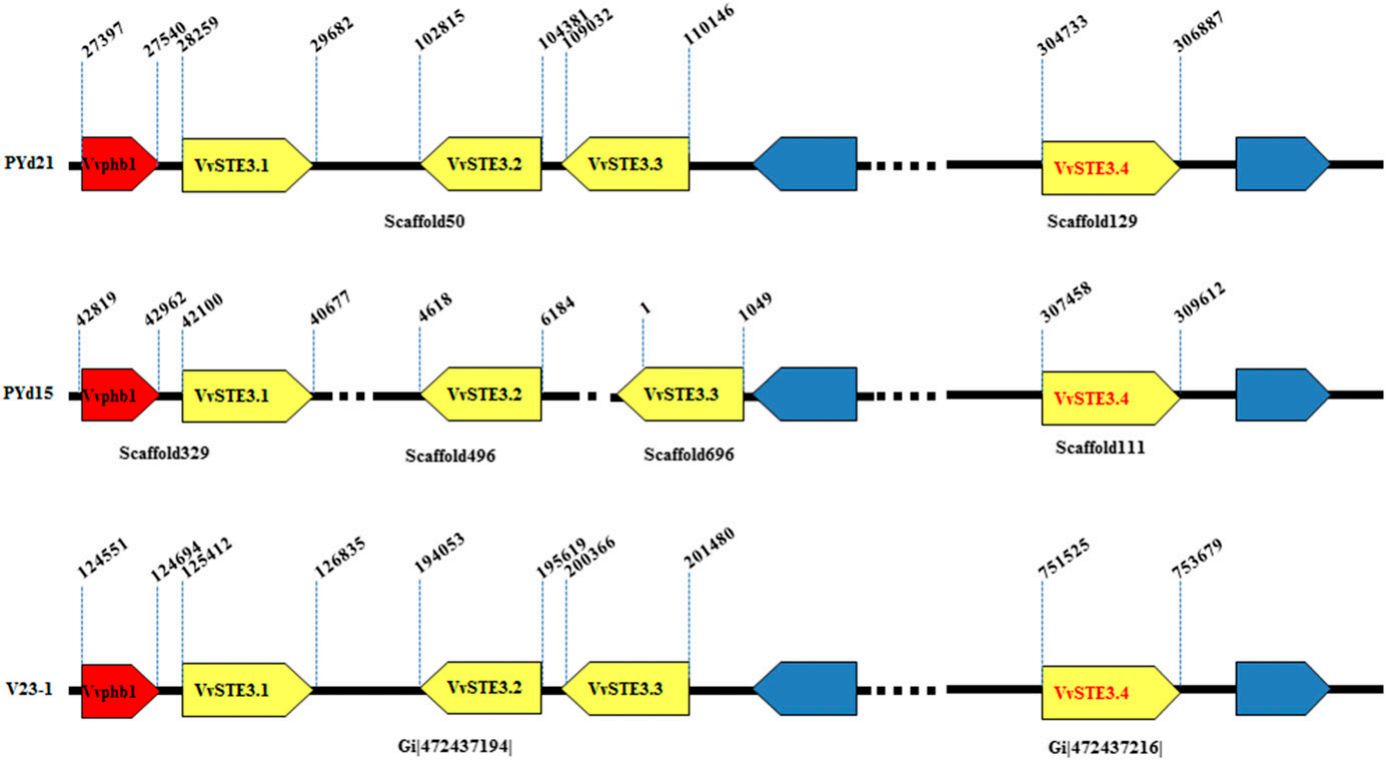
Overview of the *MAT-B* genes and pheromone receptor like genes of *V. volvacea* PYd21, PYd15, and V23-1. Pheromone precursor genes are indicated in red, pheromone receptor (like) genes in yellow (the new and fourth STE3 gene has red text), and other genes in blue. Relative positions of the genes on their corresponding genome scaffolds are indicated by black numbers showing the start and stop position of each gene. Corresponding scaffold numbers are indicated under the genes for each respective genome.

**Figure 4:**
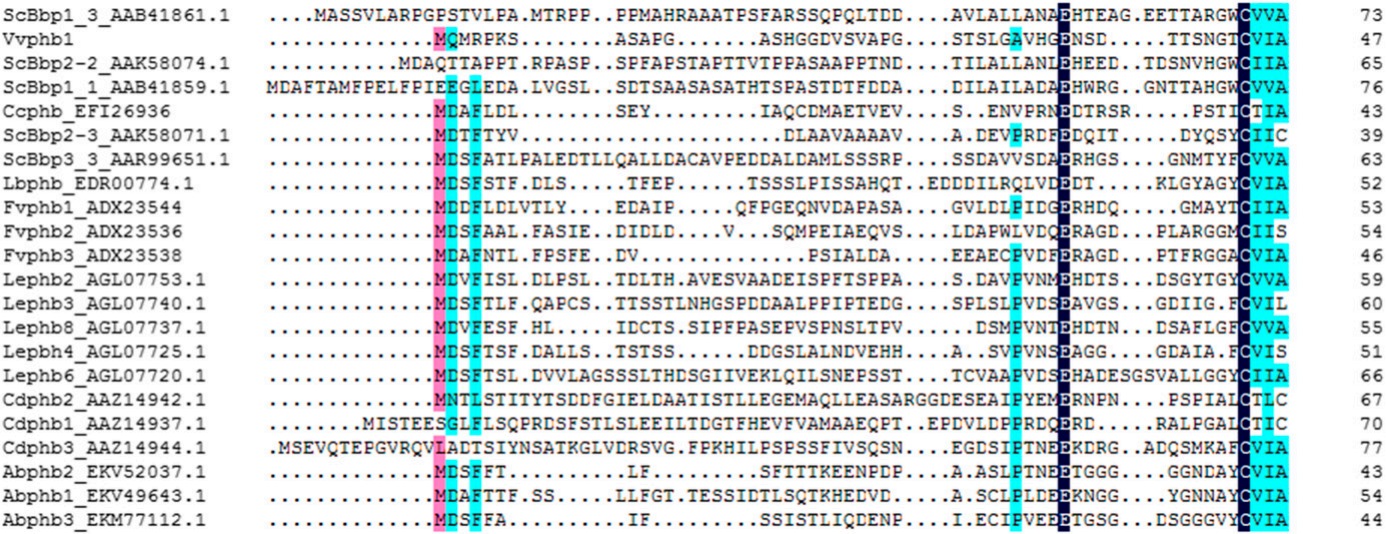
Alignments of pheromone precursor protein sequences of mushroom forming fungi *V. volvacea* (Vv), *S. commune* (Sc), *C. cinerea* (Cc), *L. bicolor* (Lb), and *F. velutipes* (Fv), showing typical conservation of the CAAX motifs, and a Glu (E) at a position ∼10 AA upstream from the CAAX motif, which are both needed for proper maturation. Conservation of AAs is indicated by shading with black (complete), purple (high), and light blue (medium).

*MAT-B* loci are typically composed of pheromone receptor and pheromone precursor genes. *V. volvacea* V23-1 had been reported to contain three pheromone receptors (*vv-rcb1*, *vv-rcb2*, and *vv-rcb3*), but no pheromone precursors (Bao *et al.* 2013). Homology searches using *C. cinerea* okayama7#130 pheromone receptors indicated four pheromone receptor genes in *V. volvacea* PYd15 and PYd21 (Figure 3, GenBank accessions: JX982139.1, JX982140.1, JX982141.1, and JX982142.1), as well as in the genome of strain V23-1. In addition, a pheromone precursor gene (*Vvphb1*) was newly found 719 bp upstream in the flanking region of pheromone receptor *VvSTE3.1* in strain PYd21 (Figure 3). Identical sequences for *Vvphb1* were detected in the genomes of PYd15 and V23-1 at the same relative position, demonstrating that *V. volvacea* in general has a complete *MAT-B* locus. Gene *Vvphb1* was predicted to encode a 47-AA-long pheromone precursor protein with a typical conserved glutamic acid (Glu, “E”) positioned ∼10 AA upstream of the C-terminal CAAX box, and can thus be expected to mature properly (Figure 4). Transcriptome data of *V. volvacea* PYd21 (Chen *et al.* 2013) furthermore confirmed that this gene was expressed, although at a low level. The pheromone receptors were named *VvSTE3.1* to *STE3.4* by analogy with other fungal STE3-like pheromone receptors, with superscripts ^–PYd15^ and ^–PYd21^ designating the strains of origin. To avoid confusion during comparisons of PYd15, PYd21 and V23-1, we adopted new names for the three previously identified V23-1 pheromone receptor genes (the previous report did not identify which gene sequences corresponded with *vv-rcb1*, *vv-rcb2*, and *vv-rcb3*). Following sequence similarities between PYd15, PYd21, and V23-1 pheromone receptors, this resulted in *VvSTE3.1*^-V23-1^, *VvSTE3.2*^-V23-1^, and *VvSTE3.3*^-V23-1^ on scaffold 24, and a new V23-1 pheromone receptor *VvSTE3.4^-V23-1^* on scaffold gi|472437216| (Figure 3). Alignment of the *VvSTE3.1* (1424 bp, 378 AA), *VvSTE3.2* (1567 bp, 447 AA), *VvSTE3.3* (1115 bp, 333 AA), and *VvSTE3.4* (2155 bp, 601 AA) pheromone receptor genes revealed 100% sequence identity between the respective homologs of PYd15, PYd21, and V23-1, except for one single nucleotide polymorphism (SNP) in *VvSTE3.4*^-PYd15^ (Figure S7, Figure S8, Figure S9, and Figure S10). Thus, no allelic variation was found in the *MAT-B* loci of the three *V. volvacea* genomes.

Prediction of transmembrane domains (TMHMM-based) indicated seven membrane-spanning regions in VvSTE3.1–VvSTE3.3, which is characteristic for pheromone receptors. VvSTE3.4 contained only five obvious membrane-spanning helices, while two more were indicated (present, but values below threshold). One of the below-threshold helices could be restored when the interrupting intron in the gene model was ignored. Transcriptome data clearly indicated that this intron was normally removed, and VvSTE3.4 was therefore unlikely to represent a normal pheromone receptor. Alignment of the respective contigs that contained the pheromone receptor genes of PYd15, PYd21, and V23-1, indicated a similar organization of the four pheromone receptors within the genomes (Figure 3).

### Identification of MAT-B genes in additional V. volvacea strains

To test for possible *MAT-B* gene polymorphism, partial pheromone receptor gene sequences were amplified from 14 SSIs that corresponded to the seven heterokaryons used for *MAT-A* analysis before (two SSIs per strain, Table 1, Figure S7, Figure S8, Figure S9, and Figure S10). Sequence similarity exceeded 97.5% for each of the pheromone receptor gene homologs, and most gene sequences were only distinguished by a few SNPs (Figure S8 and Figure S9). Notably, several SNPs in pheromone receptor *VvSTE3.2* or *VvSTE3.3* were conserved between multiple strains, and might be useful for studies of recombination between strains. The conservation of the DNA and protein sequences of the *V. volvacea* pheromone receptors supported previous observations, and pheromone receptors were not expected to participate in mating-type determination.

### Phylogenetic mapping of pheromone receptors

**Figure 5:**
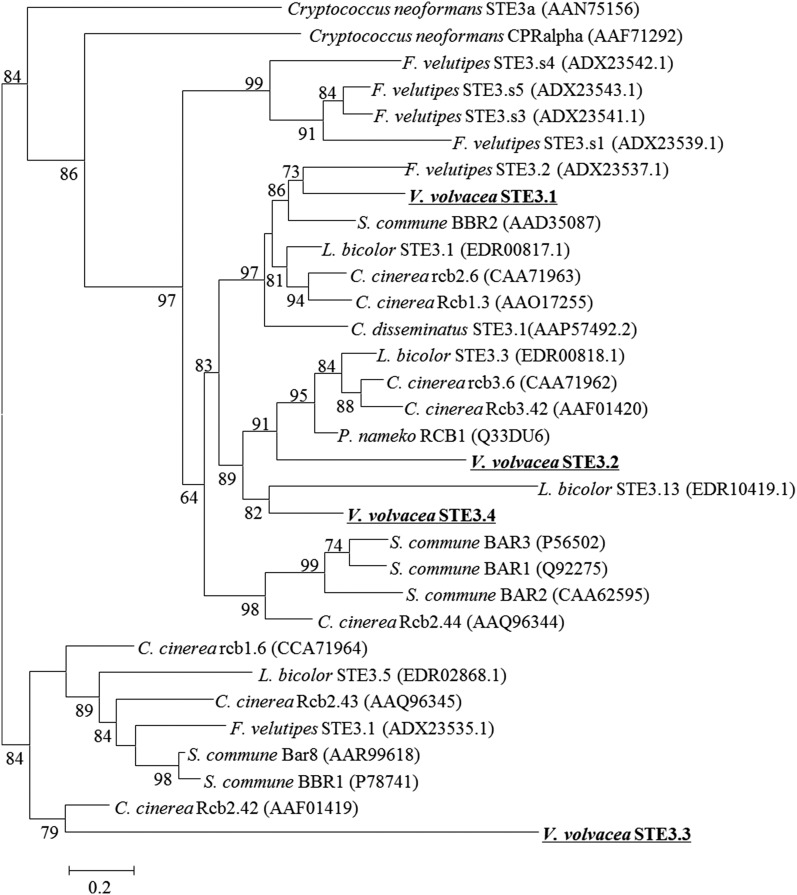
Phylogenetic tree depicting the relationship between pheromone receptor and pheromone receptor-like proteins from bipolar (*Coprinellus disseminatus* and *P. nameko*) and tetrapolar (the others) homobasidiomycetes, with that of *V. volvacea*. *Cryptococcus neoformans* was used as out-group.

Agaricomycetes with tetrapolar mating-type systems generally possess pheromone receptor genes that participate in mating type discrimination, together with closely similar genes that do not participate in mating type discrimination. The former are highly polymorphic in DNA and amino acid sequence when they discriminate mating types, while the latter are conserved between strains (May *et al.* 1999; van Peer *et al.* 2011; Kües 2015). The pheromone receptor (like) genes are usually divided over two main, ancient, lines. In an attempt to distinguish which *VvSTE3* genes might have been the actual *MAT-B* genes in absence of significant polymorphism, we performed phylogenetic mapping with known mating-type determining pheromone receptors and pheromone receptor-like proteins (Figure 5). VvSTE3.1, VvSTE3.2, and VvSTE3.4 originated from the same main clade, while VvSTE3.3 was found in the other main clade (Figure 5). VvSTE3.1 grouped closest with mating type specific pheromone receptor FvSTE3.2 of *F. velutipes*, while VvSTE3.2 and VvSTE3.4 were more closely associated with nonmating type specific pheromone receptors RCB1 of *Pholiota nameko* and STE3.13 of *L. bicolor*. VvSTE3.3 grouped nearest to mating type specific *C. cinerea* pheromone receptor RCB2.42. Grouping is no absolute indication for mating type specificity. However, the presence of a pheromone precursor next to gene *VvSTE3.1*, together with the close association of VvSTE3.1 with a mating type specific pheromone receptor, both suggested that this pheromone receptor has had a function in *B* mating type discrimination before the mating type locus became redundant.

### MAT-A loci control heterokaryotization

Polymorphic *MAT-A* gene sequences indicated *MAT-A* controlled mating type discrimination and mating. To test this, homokaryons of the eight different *A* mating-type groups were crossed in all possible combinations (Figure 2). Single hyphae of the resulting colonies from each cross were subcultured and analyzed using *MAT-A* allele specific primers to distinguish homokaryons from heterokaryons (primers; Table S1B). Except for the combinations of mating type A8 with A4 and A5, each of the eight *A* mating-types was compatible with the other seven (Table 1 and Figure 2). A8, although containing an incomplete HD2 protein, combined properly with A1, A2, A3, A6, and A7. This suggested that HD2 proteins of A4 and A5 are either incompatible with the HD1 of A8, or that their expression/function might have been compromised. Overall, the results clearly demonstrated the necessity of specific *A* mating type combinations for the development of heterokaryons in *V. volvacea*. Various combinations of putative pheromone receptor genes (*VvSTE3.2* and *VvSTE3.3*) with different SNPs indicated no relation to heterokaryon formation (not shown).

### Analysis of karyotypes

The basidia of *V. volvacea* have been described as mainly tetrasporic, yet can also be bisporic, trisporic, and pentasporic (Chang and Yau 1971; Li 1982; Li and Chang 1991; Xie *et al.* 2010). Reported percentages mentioned 9.06% bisporic, 21.01% trisporic, and 69.93% tetrasporic basidia, corresponding with estimated percentages of 7.23% heterokaryotic spores, and 92.77% homokaryotic spores. Homokaryotic spores included 89.16% mononuclear and 3.61% binuclear spores (Li and Chang 1991; Xie *et al.* 2010; Bao *et al.* 2013). To determine if, and possibly which, percentages of *V. volvacea* spores were homokaryotic or heterokaryotic, karyotyping was performed with molecular markers.

A screen of 112 SSIs (F1) from strain PY1 using two SCAR markers (SCAR15 and SCAR48, Table S2, showed even distribution of the four marker bands—each marker generated a smaller or a larger band for karyotype PYd15 or PYd21, Figure S11). Recombination between SCAR15 and SCAR48 markers was even (Table S2). Three SSIs contained double bands for both SCAR markers, suggesting heterokaryotic spores. Five and two SSIs showed double bands for only one SCAR marker (5 × SCAR15, and 2 × SCAR48), indicating binuclear spores with different copies of only one marker due to meiotic recombination prior to nuclear distribution over the spores. Overall, the number of possible heterokaryotic spores (10) was low compared to the number of homokaryotic (102) spores (<10%), in correspondence with microscopically determined ratios (Li and Chang 1991; Xie *et al.* 2010).

Analysis of 132 SSIs from heterokaryon H1521 (PYd15 × PYd21) with SCAR15 and SCAR48 confirmed similar distribution and recombination patterns (Table S3). Surprisingly, distribution of the third marker that was included in this screen (SCAR1270) showed 37 PYd15, and 78 PYd21 specific bands (*i.e.*, 1:2 or possibly a 1:3 ratio, Table S3). Recombination between the alleles represented by the three markers was even (50%, Table S3). Possible heterokaryons were indicated by one SSI that contained double bands for all three markers, two SSIs that contained double bands for two markers. An unexpected high number of 22 SSIs contained double bands for only one marker (SCAR15 5 ×, SCAR48 12 ×, SCAR1270 5 ×). Possible heterokaryotic spores (25 in total if assuming all SSIs with a double band were heterokaryons) represented 18.9% of the spore total, which was higher than the expected number, and the results with only two markers in PY1 (< 10%).

Recent identification of SV markers for PYd15 and PYd21 (Wang *et al.* 2015) enabled analysis of homo- and heterokaryosity with a higher resolution. Twenty-four different SV markers of 10 independent linkage groups were selected to analyze 105 SSIs of heterokaryon H1521, together with *MAT-A* loci specific primers for A1 and A2 mating types (Table S4).

*MAT-A* loci were distributed evenly over the SSIs (46 SSIs A1, 49 SSIs A2). Eight of the 105 SSIs contained two (both A1 and A2) *MAT-A* loci. Two SSIs did not contain *MAT-A* loci (Table S4). Together, this suggested eight heterokaryotic spores (double *MAT-A* loci), 95 homokaryotic spores (one *MAT-A* locus), and two undetermined spores (no *MAT-A* loci). Heterokaryosity of six of the eight SSIs with two *MAT-A* loci was supported by multiple doubled SV markers. SSI no. 22 had 19 SV-doubles, SSI no. 104 had 14 SV-doubles, SSI no. 95 had 11 SV-doubles, SSI no. 70 had 10 SV-doubles, SSI no. 88 had nine SV-doubles, and SSI no. 81 had seven SV-doubles. SSI no. 51 and SSI no. 43, while having two *MAT-A* loci, contained only four and zero doubled SV markers respectively.

Conversely, from the nine SSIs with the highest number of doubled SV markers, two did not contain double *MAT-A* loci. Moreover, one of these nine SSIs did not contain any *MAT-A* locus (Table S4). The presence of two *MAT-A* loci seems therefore indicative, but not conclusive as a measure for heterokaryosity of *V. volvacea* spores.

The total number of SSIs with one or more doubled SV markers (53, or 49.5%) far exceeded the estimated 7.23% percent of heterokaryotic spores (Li and Chang 1991; Xie *et al.* 2010), while the number of SSIs with few doubled SV markers (only one, two, or three, in total 37) was much higher than for SSIs with more doubled SV markers (Table S4). Finally, 10 of the 24 SV markers approximated a 2:1 or 3:1 (1:2 or 1:3, respectively) distribution, instead of a normal 1:1 distribution (Table S4). The uneven distribution of almost half of the analyzed alleles indicated an additional genetic mechanism with substantial influence on the karyotypes in addition to the bipolar and secondary homothallic mechanisms.

### Fruiting ability of V. volvacea strains

**Figure 6:**
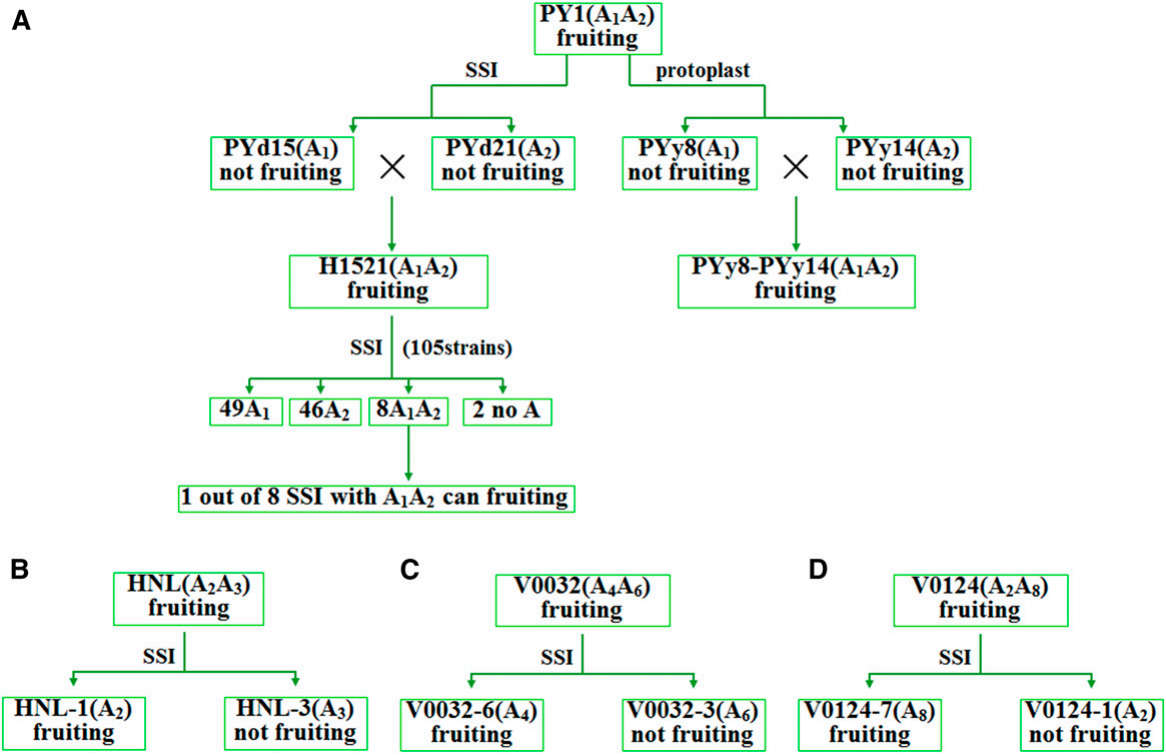
Overview of fruiting assays with heterokaryotic and homokaryotic *V. volvacea* strains. Mating types are indicated when known, origin of homokaryons (protoplast derived or SSI-based) is indicated by lines connecting with their parental heterokaryons. (A) Fruiting ability of PY1 and PY1 derived homokaryons PYd15, PYd21, PYy8, PYy14, and newly crossed heterokaryons H1521 and PYy8-PYy14. (B**–**D) Fruiting ability of the *V. volvacea* wild strains and SSIs whose mating types were analyzed in this study.

A collection of independent mushroom cultivation tests of heterokaryotic strains PY1 (cultivar), H1521 (derived from PY1), HNL (wild), V0032 (wild), V0124, and multiple homokaryons (SSI or protoplast based) was compiled into an overview of fruiting behavior of homo- and heterokaryotic strains (Figure 6). PY1 is a commercial strain from Fuzhou (Fujian, China) that is well known for its ability to produce regular and normal fruiting bodies. Homokaryotic strains PYd15 (sequenced) and PYd21 (sequenced) were obtained as SSIs from PY1, and PYy14 and PYy8 were obtained from protoplasts of PY1. Each of these four homokaryons had a single *MAT-A* locus (Figure 6), and was incapable of producing fruiting bodies. When crossed, heterokaryons H1521 (PYd15 × PYd21) and PYy14-PYy8 (PYy14 × PYy8) both produced normal fruiting bodies. These results suggested a typical bipolar mating type system.

The SSIs from strain H1521 that contained double *MAT-A* loci [(A1 + A2), Table S4 and Figure 6] showed varying fruiting ability. While SSI H1521-104 (*MAT-A*, A1A2) produced mature fruiting bodies, the other seven SSIs with two *MAT-A* loci did not. This indicated that fruiting body formation of self-fertile SSIs did not strictly relate to the presence of two compatible *MAT-A* loci. Analyses of wild isolates further confirmed these results. Heterokaryotic strains V0032, V0124, and HNL produced normal fruiting bodies. Some of their SSIs could develop normal fruiting bodies while clearly having only a single *MAT-A* locus (*e.g.*, V0124-7), some developed fruiting bodies that were arrested during maturation [*e.g.*, HNL-1, V0032-6, usually arrested in the Egg stage (stage as defined by Li 1982)], and others, did not produce fruiting bodies at all (*e.g.*, HNL-3 and V0032-6).

Together, this data strongly suggested that additional factors, other than *MAT-A* or homo- and heterokaryosity influenced fruiting and mushroom development in *V. volvacea*.

### Expression of MAT-A and MAT-B genes in homokaryons and heterokaryons

*MAT-A* genes seem to control mating in *V. volvacea*, yet generation of typical dikaryotic hyphae with clamps and paired nuclei are absent. Interestingly, gene expression analysis of *MAT-A* and *MAT-B* genes in colonies of PYd15, PYd21, heterokaryon H1521, and in primordia formed by H1521, revealed large differences in expression levels. Expression of *MAT-A* A1 genes (*vv-HD1*^–PYd15^ and *vv-HD2*^–PYd15^) was low in both homokaryons (PYd15 and PYd21) and in the heterokaryon (H1521), but was increased in primordia of H1521. Meanwhile, *MAT-A* A2 genes (*vv-HD1*^–PYd21^ and *vv-HD2*^–PYd21^) showed very high expression in the PYd21 homokaryon, but virtually no expression in the dikaryon and in the primordia of H1521 (Table S5 and Figure S12). Thus, *MAT-A* A1 genes were heterokaryon-specific, while *MAT-A* A2 genes were homokaryon-specific in a cross with PYd15 × PYd21. *MAT-B* genes, especially *VvSTE3.1* and *VvSTE3.4*, were expressed more strongly in the primordia than in the homokaryons (Table S5 and Figure S12). Moreover, *VvSTE3.4* was also expressed in homokaryon PYd15, but not in PYd21, showing mating type specific expression of *MAT-B* as well. Clearly, *MAT-A* and *MAT-B* genes were regulated differently than in a regular bipolar mechanism, which could well be related to the absence of clamps and multi nuclear hyphae in *V. volvacea*.

## Discussion

The life cycle and mating type system of *V. volvacea* has long been a subject of debate. Chang and Yan (1971), as well as others (Elliot 1982; Royse *et al.* 1987) had previously classified *V. volvacea* as primarily homothallic, based on observations of SSIs that were self-fertile and could produce fruiting bodies. Homo- or monokaryotic fruiting has been known to occur in heterothallic fungi as well, mostly as a result of stress, but typically exhibits low fruiting frequencies (*e.g.*, Stahl and Esser 1976; Esser and Meinhardt 1977; Esser *et al.* 1979). The high incidence of self-fertile SSIs in *V. volvacea* (*e.g.*, 76% in Chang and Yan 1971) therefore suggested a mechanism other than just a heterothallic life cycle. The inability of substantial percentages of SSIs to produce fruiting bodies (24% in Chang and Yan 1971) had remained unexplained. Recently, an exploratory study of the *V. volvacea* mating type genes suggested that, instead of being homothallic, *V. volvacea* was pseudo or secondary homothallic (Bao *et al.* 2013). This was based on the findings that the *MAT-B* locus was incomplete (no pheromone precursor genes), and not mating-type-specific, while the *MAT-A* locus was mating-type-specific, and only heterokaryotic SSIs (determined with *MAT-A* specific markers) could produce fruiting bodies (Bao *et al.* 2013).

In this study, we analyzed the mating type system of *V. volvacea* in more detail and in multiple strains. We further explored possible homothallism as well as secondary homothallism (heterokaryotic spores), using multiple markers for karyotyping in combination with fruiting assays. Our data clearly indicated that *V. volvacea* is neither primarily homothallic, nor strongly secondary homothallic, but instead employs a mixture of genetic systems.

The mating type system of *V. volvacea*, s*ensu stricto*, is bipolar, and seemed to have been derived from a tetrapolar mating-type system like in several other bipolar Agaricomycetes (Aimi *et al.* 2005; James *et al.* 2006, 2011). The *MAT-B* locus was still complete with a pheromone receptor (*VvSTE3.1*) and a pheromone precursor (*Vvphb1*). However, high conservation (>97.5%) of pheromone receptor and pheromone precursor gene sequences between mating compatible strains showed that *MAT-B* was no longer mating type specific. Identification and analysis of seven *MAT-A* loci, in addition to the previously identified *MAT-A* locus of strain V23-1, indicated that every *MAT-A* locus contained a single, mating-type-specific pair of HD1 and HD2 genes. Crossing of different strains clearly demonstrated that only strains with different *MAT-A* loci could generate heterokaryons. The strict control over mating by the *MAT-A* locus of *V. volvacea* was further emphasized by the inability of mating types with an incomplete HD2 protein (mating type A8), to form heterokaryons with mating type A4 or A5 (Table 1). Regular heterokaryotization, as controlled by *MAT-A*, does seem to contribute to the ability to produce mushrooms, in agreement with the observations of Bao *et al.* (2013). First of all, heterokaryons from crossed, self-sterile, homokaryons are able to generate fruiting bodies (Figure 6), and, second, strains isolated from the wild are often heterokaryotic (*e.g.*, HNL, V0032, V0124, V0076, and V0077), which might suggest an advantage of hetero- over homokaryotic mushrooms. In this respect, *V. volvacea* clearly fits a bipolar heterothallic system.

The conclusion that fruiting of SSIs could be explained by a secondary homothallic system (and required heterokaryotic spores with both *MAT-A* loci) instead of a homothallic system (Bao *et al.* 2013), was demonstrated to be incorrect, or, more precisely, incomplete. The existence of a secondary homothallic life cycle in *V. volvacea* was supported by bi- (9.06%) and trisporic (21.01%) basidia, a possible ratio of binuclear spores (∼10.8%) (Li and Chang 1991; Xie *et al.* 2010), and *MAT-A* and SV-marker-based analysis of SSIs that indicated heterokaryotic spores (Table S4). We do therefore expect a bipolar as well as a secondary homothallic life cycle in *V. volvacea*.

However, multiple SSIs with only one *MAT-A* locus were found to produce fruiting bodies (Figure 6), meaning that secondary homothallism does not account for all fruiting SSIs. This was further indicated by the inconsistency between reported, high numbers (76%) of SSIs that could produce fruiting bodies (Chang and Yan 1971), and estimated low numbers (7–11%, 18.6%) of heterokaryotic spores (Li and Chang 1991; Xie *et al.* 2010; Bao *et al.* 2013).

Our analysis of SSIs with *MAT-A* A1 and A2 specific markers, together with SV markers, indicated the existence of spores that were neither strictly homo-, nor strictly heterokaryotic. Some of the clearest examples were H1521 SSI no. 80, which contained no *MAT-A* loci but seven doubled SV markers, and SSI no. 43, which contained two *MAT-A* loci, but no doubled SV markers (Table S4). In addition, SV markers showed normal (50%) recombination (Table S4), while the vast majority of the SSIs contained only few (one, two, or three) doubled SV markers out of a total of 24 SV markers. The total number of SSIs that contained one or more doubled SV markers, 53 (or 49.5%), was also much higher than would be expected based upon the estimated ∼10.8% of heterokaryotic spores (Li and Chang 1991; Xie *et al.* 2010). This suggested that not all SSIs that contained a doubled SV marker were also heterokaryotic. Another surprise was the 2:1 or 3:1 (1:2 or 1:3, respectively) distribution ratio of 10 of the 24 SV markers (42%), as opposed to normal 1:1 ratios.

Aneuploidy in *V. volvacea* had been rejected previously by Chiu and Moore (1999; discussed in Li *et al.* 1992; Chiu 1993). Besides, doubling of the SV markers in SSIs seemed to occur independently of the respective linkage groups (*i.e.*, often only one marker of a group was doubled). However, partial aneuploidy (loss or gain of a part of a chromosome) could help to explain the varying presence of SV markers in SSIs independently of their linkage groups. Partial aneuploidy might also help to explain the wide range of phenotypic variations of progenies derived from a single meiotic spore (Li and Chang 1979; Chang *et al.* 1981; Chang and Li 1991; Li and Chang 1991), as well as the irregular capacity of SSIs to produce fruiting bodies.

Interestingly, only four of the SV markers (SV416 in SV linkage group 1; SV109 in SV linkage group 5; SV419 in SV linkage group 8, and SV010 in SV linkage group 9), were unequally distributed (2:1, 3:1, or 1:2, 1:3, respectively) within the subgroup of SSIs that did not contain doubled SV markers. This indicated a possible relationship between doubling of SV markers and 2:1 or 3:1 (1:2, 1:3, respectively) distribution ratios. Other explanations for unequally distributed SV markers could be intragenomic conflicts, such as the localization of a marker on a low-recombination region of a chromosome (*e.g.*, near a centromere, Lyttle 1991), killer loci, or synthetic lethality. It is unlikely that all 24 SV markers would be located near a centromere. However, selfish gene models where one allele causes a biased segregation due to killing off one or more of the other spores formed on the basidium, or during germination, or a gene interaction model where certain combinations of alleles are detrimental, and thus never observed in spore-derived colonies, are of course possible.

Taken together, we conclude that *V. volvacea* is capable of bipolar, secondary homothallic, and homothallic life cycles, of which the bipolar mechanism seems to be the most abundant. Genetic content is distributed unequally over a substantial number of spores, which might be caused by partial aneuploidy, in combination with another mechanism. It will be very interesting to test if, and to what extent, partial aneuploidy causes the observed irregular distribution of gene content, and which other possible mechanisms might contribute to these observations.

## Supplementary Material

Supplemental Material
