# Supplementary material for: Fruiting Body Formation in Volvariella volvacea Can Occur Independently of Its MAT-A-Controlled Bipolar Mating System, Enabling Homothallic and Heterothallic Life Cycles
Source: G3 (Bethesda). 2016 May 16;6(7):2135–46. doi: 10.1534/g3.116.030700 (PMC4938666; doi:10.1534/g3.116.030700)
Supplement: Supplemental Material [file supp_6_7_2135__index.html]

Fruiting Body Formation in Volvariella volvacea Can Occur Independently of Its MAT-A-Controlled Bipolar Mating System, Enabling Homothallic and Heterothallic Life Cycles — Supplemental Material 

# Fruiting Body Formation in *Volvariella volvacea* Can Occur Independently of Its *MAT-A*-Controlled Bipolar Mating System, Enabling Homothallic and Heterothallic Life Cycles

## Supplemental Material for Chen *et al.*, 2016

**Files in this Data Supplement:**

- File S1 - File contains legends for all Supplemental Tables and Figures. (.pdf, 107 KB)
- Table S1 - PCR primers used for: (A) cloning of *MAT-A* and *MAT-B* loci, (B) *MAT-A* locus identification in mating tests, (C) qRT-PCR experiments for gene expression analysis, and (D) SCAR marker analysis. (.pdf, 27 KB)
- Table S2 - (.xls, 33 KB)
- Table S3 - Karyotype analysis of 112 single spore isolates from *V. volvacea* strain PY1 using markers SCAR15 and SCAR48. (.xls, 53 KB)
- Table S4 - Karyotype analysis of 105 single spore isolates from *V. volvacea* strain H1521 using 24 structural variance (SV) markers of 10 different linkage groups. (.xlsx, 36 KB)
- Table S5 - The expression level of *MAT-A* and *MAT-B* genes in different stages of *V. volvacea*. (.xlsx, 10 KB)
- Figure S1 - Alignment of *V. volvacea vv-hd1* gene DNA sequences. (.tif, 1,524 KB)
- Figure S2 - Alignment of *V. volvacea* VV-HD1 proteins. (.tif, 7,211 KB)
- Figure S3  - Alignment of *V. volvacea vv-hd2* gene DNA sequences. (.tif, 1,352 KB)
- Figure S4 - Alignment of *V. volvacea* VV-HD2 proteins. (.tif, 6,890 KB)
- Figure S5 - Genome sequences of regions containing the *MAT-A* loci of PYd21, PYd15 and V23-1 indicating the conserved positions of primers LP-f and LP-r (black dotted boxes), relative to the respective HD1 and HD2 genes of which the start and end positions are indicated in red (PYd15), blue (PYd21) and purple (V23-1). (.tif, 4,393 KB)
- Figure S6 - Restriction enzyme fragment length polymorphism analysis of PCR products of different *MAT-A* loci of *V. volvacea*. (.tif, 488 KB)
- Figure S7 - Alignment of *V. volvacea Vv-STE3.1* gene DNA sequences. (.tif, 982 KB)
- Figure S8 - Alignment of *V. volvacea Vv-STE3.2* gene DNA sequences. (.tif, 1,340 KB)
- Figure S9 - Alignment of *V. volvacea Vv-STE3.3* gene DNA sequences. (.tif, 2,641 KB)
- Figure S10 - Alignment of *V. volvacea Vv-STE3.4* gene DNA sequences. (.tif, 1,562 KB)
- Figure S11 - Figure representing SCAR and SV marker analysis based gel electrophoresis results as used for all discussed marker analyses. (.tif, 347 KB)
- Figure S12 - (A) Expression levels of *MAT-A* genes in the homokaryon (PYd21, PYd15), the dikaryon H1521 (cross of PYd21 with PYd15) and the primordia (generated from strain H1521). (.tif, 94 KB)
