# Supplementary material for: Fruiting Body Formation in Volvariella volvacea Can Occur Independently of Its MAT-A-Controlled Bipolar Mating System, Enabling Homothallic and Heterothallic Life Cycles
Source: G3 (Bethesda). 2016 May 16;6(7):2135–46. doi: 10.1534/g3.116.030700 (PMC4938666; doi:10.1534/g3.116.030700)
Supplement: Supplemental Material [file supp_g3.116.030700_FileS1.pdf]

Supplemental Information

Supplemental Tables

Table S1 PCR primers used for: (**A**) cloning of *MAT-A* and *MAT-B* loci, (**B**) *MAT-A* locus identification in mating tests, (**C**) qRT-PCR experiments for gene expression analysis, and (**D**) SCAR marker analysis.

Table S2 Karyotype analysis of 112 single spore isolates from *V. volvacea* strain PY1 using markers SCAR15 and SCAR48. Distribution of SCAR15 PYd15 (“0”) and PYd21 (“1”) alleles is shown in column B, distribution of SCAR48 PYd15 (“0”) and PYd21 (“1”) alleles is shown in column C. Totals are summarized under each column. Recombination between alleles is indicated in column D. Corresponding SV marker linkage groups (Wang *et al.* 2015) in which SCAR markers were located based on the genome sequences are indicated after each SCAR marker name.

Table S3 Karyotype analysis of 132 single spore isolates from *V. volvacea* strain H1521 using markers SCAR15, SCAR48 and SCAR1270. Distribution of SCAR15 PYd15 (“0”) and PYd21 (“1”) alleles is shown in column B, distribution of SCAR48 PYd15 (“0”) and PYd21 (“1”) alleles is shown in column C. Distribution of SCAR15 PYd15 (“0”) and PYd21 (“1”) alleles is shown in column D. Totals are summarized under each column. Recombination between respective alleles is indicated in columns E, F and G. Corresponding SV marker linkage groups (Wang *et al.* 2015) in which SCAR markers were located based on the genome sequences are indicated after each SCAR marker name.

Table S4 Karyotype analysis of 105 single spore isolates from *V. volvacea* strain H1521 using 24 structural variance (SV) markers of 10 different linkage groups.

Markers and corresponding linkage groups are indicated with numbers corresponding to Wang *et al.* (2015) above each column. *A* mating types as determined with specific primers are indicated for each SSI. Presence of the PYd15 specific allele is indicated with “0”, PYd21 with “1”, no band for either of the two alleles with “2” and both alleles with “3”. Distributions for each SV marker are summarized at the bottom of each column. Significance of found allele distributions was tested with hypotheses (1:1, 1:2, 1:3, 3:1, 2:1) according to χ^2 test, and results are indicated with “y” (significant) or “n” (significance for this distribution statistically not supported). Totals of doubled or missing SV markers (as numbers and percentages of a total of 24) are given in the last three columns.

Table S5 The expression level of *MAT-A* and *MAT-B* genes in different stages of *V. volvacea*.

Supplemental Figures

Figure S1 Alignment of *V. volvacea vv-hd1* gene DNA sequences. Originating strains for the respective sequences are indicated left of the sequence. Names of strains with identical sequences are separated by “/”. Conservation of base pairs is indicated by shading with black (complete), purple (high) and light blue (medium).

Figure S2 Alignment of *V. volvacea* VV-HD1proteins. Originating strains for the respective sequences are indicated left of the sequence. Names of strains with identical sequences are separated by “/”. Dimerization motifs are indicated with (Di, red boxes), homeodomain 1 domains with (HD1, blue box), and nuclear localization signals with (NLS, black boxes). Conservation of amino acids is indicated by shading with black (complete), purple (high) and light blue (medium).

Figure S3 Alignment of *V. volvacea vv-hd2* gene DNA sequences. Originating strains

for the respective sequences are indicated left of the sequence. Names of strains with identical sequences are separated by “/”. Conservation of base pairs is indicated by shading with black (complete), purple (high) and light blue (medium).

Figure S4 Alignment of *V. volvacea* VV-HD2 proteins. Originating strains for the respective sequences are indicated left of the sequence. Names of strains with identical sequences are separated by “/”. Dimerization motifs are indicated with (Di, red boxes), homeodomain 1 domains with (HD1, blue box), and nuclear localization signals with (NLS, black boxes). Note the absence of a complete HD2 domain in strain V0124-7 (mating type A8). Conservation of amino acids is indicated by shading with black (complete), purple (high) and light blue (medium).

Figure S5 Genome sequences of regions containing the *MAT-A* loci of PYd21, PYd15 and V23-1 indicating the conserved positions of primers LP-f and LP-r (black dotted boxes), relative to the respective HD1 and HD2 genes of which the start and end positions are indicated in red (PYd15), blue (PYd21) and purple (V23-1).

Figure S6 Restriction enzyme fragment length polymorphism analysis of PCR products of different *MAT-A* loci of *V. volvacea*. Restriction enzymes are indicated on the left, homokaryons and corresponding mating types that were identified after sequencing are indicated above the restriction patterns.

Figure S7 Alignment of *V. volvacea Vv-STE3.1* gene DNA sequences. Complete genes are shown for the three genomes (PYd15, PYd21 and V23-1), partial sequences are shown for the additionally sub cloned *Vv-STE3.1* genes. Originating strains for the respective sequences are indicated left of the sequence. Names of strains with identical sequences are separated by “/”. Conservation of base pairs is indicated by shading with black (complete), purple (high) and light blue (medium).

Figure S8 Alignment of *V. volvacea Vv-STE3.2* gene DNA sequences. Complete

genes are shown for the three genomes (PYd15, PYd21 and V23-1), partial sequences are shown for the additionally sub cloned *Vv-STE3.2* genes. Originating strains for the respective sequences are indicated left of the sequence. Names of strains with identical sequences are separated by "/". Conservation of base pairs is indicated by shading with black (complete), purple (high) and light blue (medium).

Figure S9 Alignment of *V. volvacea Vv-STE3.3* gene DNA sequences. Complete genes are shown for the three genomes (PYd15, PYd21 and V23-1), partial sequences are shown for the additionally sub cloned *Vv-STE3.3* genes. Originating strains for the respective sequences are indicated left of the sequence. Names of strains with identical sequences are separated by "/". Conservation of base pairs is indicated by shading with black (complete), purple (high) and light blue (medium).

Figure S10 Alignment of *V. volvacea Vv-STE3.4* gene DNA sequences. Complete genes are shown for the three genomes (PYd15, PYd21 and V23-1), partial sequences are shown for the additionally sub cloned *Vv-STE3.4* genes. Originating strains for the respective sequences are indicated left of the sequence. Names of strains with identical sequences are separated by "/". Conservation of base pairs is indicated by shading with black (complete), purple (high) and light blue (medium).

Figure S11 Figure representing SCAR and SV marker analysis based gel electrophoresis results as used for all discussed marker analyses. Shown are results for SCAR15 and SCAR21 used to determine the karyotypes of SSIs from *V. volvacea* strain PY-1. (**A**) Individual small and large bands representing presence of single alleles corresponding to PYd15 or PYd21, and double bands representing presence of both alleles in a single spore isolate (SSI). (**B**) Individual small and large bands representing presence of single alleles corresponding to PYd15 or PYd21, and double bands representing presence of both alleles in a SSI. "M"

indicates the DL2000 DNA Marker. Total absence for a marker in SSIs was interpreted as “undetermined”.

Figure S12 (A) Expression levels of *MAT-A* genes in the homokaryon (PYd21, PYd15), the dikaryon H1521 (cross of PYd21 with PYd15) and the primordia (generated from strain H1521). PYd21 *vv-HD1* and *vv-HD2* genes show homokaryon specific expression, while PYd15 *vv-HD1* and *vv-HD2* genes show primordia specific expression. (B) Expression levels of *MAT-B* genes in the homokaryon (PYd21, PYd15), the dikaryon H1521 (cross of PYd21 with PYd15) and the primordia (generated from strain H1521). Pheromone receptor genes are upregulated in the primordia, and PYd15 *VvSTE3.4* is up-regulated in the PYd15 homokaryon as well, indicating strain specific expression next to stage specific expression.
