# Supplementary material for: Fruiting Body Formation in Volvariella volvacea Can Occur Independently of Its MAT-A-Controlled Bipolar Mating System, Enabling Homothallic and Heterothallic Life Cycles
Source: G3 (Bethesda). 2016 May 16;6(7):2135–46. doi: 10.1534/g3.116.030700 (PMC4938666; doi:10.1534/g3.116.030700)
Supplement: Supplemental Material [file supp_g3.116.030700_TableS1.pdf]

Table S1 PCR primers used for: (**A**) cloning of *MAT-A* and *MAT-B* loci, (**B**) *MAT-A* locus identification in mating tests, (**C**) qRT-PCR experiments for gene expression analysis, and (**D**) SCAR marker analysis.

| Primer | Sequence |
|---|---|
| (**A**) | |
| LP-f | TCCTTAGTGCTGTCTTATCGGA |
| LP-r | CTCATCATTCTTGCCACCCTAT |
| VvSTE3.1-f | TTCTTATGGTGCGGGTTAG |
| VvSTE3.1-r | CTCGTTCATTGTGGTATTCG |
| VvSTE3.2-f | TCTTCTCCTTTCTCGGTTTCTT |
| VvSTE3.2-r | ACTATGTCGTGACTGGTCTGGT |
| VvSTE3.3-f | CCTGAACCAACTCTAAGC |
| VvSTE3.3-r | ACATCTCCAGCACCAATC |
| VvSTE3.4-f | AAAGCGACAGTAATCAGCAAA |
| VvSTE3.4-r | CCTACCCGAATACACGAAAG |
| (**B**) | |
| PYd15-f | TCAGCAAACATAACCACA |
| PYd15-r | TAAATGCCCACTCATACCC |
| PYd21-f | TGGCGTTGTCTTGATTTGC |
| PYd21-r | TGCCGAGTTCTATCTAGGTTTCT |
| HNL-3-f | GTGTGGCGTTAGCGTTAGTAGT |
| HNL-3-r | GTGGTATTGGAAGTGAAAGTGG |
| V0032-6-f | GAAGTGGTGCGGGGGTAGA |
| V0032-6-r | GCATTGATGGCGGAGTGTG |
| V0077-1-f | CACTCATAAGCCCTGTTGTTAG |
| V0077-1-r | CCACCCGTATTGTTGTTCAT |
| V0076-1-f | GGAGGTAGAGGGTTCGTGTAA |
| V0076-1-r | CGAGTAAGTCCAGGGTTTCAG |
| V0076-7-f | TCACATTGTTGCCGTTGG |
| V0076-7-r | TGTCGTCTTGGTTGGTTCG |
| V0124-7-f | TGACGGAAAGGCAATACGG |
| V0124-7-r | CCCAGAGAGTTCGTTTGATTTG |
| (**C**) | |

| | |
|---|---|
| qPCR-VvSTE3.1-f | CTTACGATTGGAGTCAGCGTTGGGA |
| qPCR-VvSTE3.1-r | TGATGTCTGTTCTTATGGTGCGGG |
| qPCR-VvSTE3.2-f | GCGAGTTGCGTTTGGTTTTG |
| qPCR-VvSTE3.2-r | GTCTTCTTTGCCTTCTTCGGATTT |
| qPCR-VvSTE3.3-f | TGCTGATAGGGCGGTCTGATGC |
| qPCR-VvSTE3.3-r | GTCCGTATTCCAGTGTGGTGCG |
| qPCR-VvSTE3.4-f | ATAAAGGTTGTAAGGGGTGCTGA |
| qPCR-VvSTE3.4-r | GATGATGGGTGTTCCTCTTGCTC |
| qPCR-GAPDH-f | CATCTTCCACTGGTGCGGCTAAG |
| qPCR-GAPDH-r | GGCTTCTCAAGGCGAACGACAA |
| qPCR-PYd21-HD1-f | GTTGTGAAGATTAGCGAGAAGCCATT |
| qPCR-PYd21-HD1-R | TTGACGACGAGTTAATGATGCTGGT |
| qPCR-PYd21-HD2-F | TCCAAGGTATTGAGCGGCACAGA |
| qPCR-PYd21-HD2-R | CGGCAGAAGGGTAGGCATTAGACT |
| qPCR-PYd15-HD1-F | GAAGTTGGGTATGAGTGGGCATTTAC |
| qPCR-PYd15-HD1-R | AAGGTGGCGGCGTCGTTAA |
| qPCR-PYd15-HD2-F | ACCCTCTTTCTTAGCCCGATTGC |
| qPCR-PYd15-HD2-R | CACGCCATTGCTTGAGAACTACTTC |
| (**D**) | |
| SCAR15-F | TGGAGGGTGTTCCTATGGGTAAA |
| SCAR15-R | GGTGTTGGCGAACAAATTCTTAG |
| SCAR48-F | GTGTGCCCCAAGTGGAAGAA |
| SCAR48-R | GTGTGCCCCATACTCCCATATGA |
| SCAR1270-F | GGCGTATGGTGATGAAGTCAACT |
| SCAR1270-R | GGCGTATGGTCCTATTTTAAGTT |
