# Supplementary figures and images for: Fruiting Body Formation in Volvariella volvacea Can Occur Independently of Its MAT-A-Controlled Bipolar Mating System, Enabling Homothallic and Heterothallic Life Cycles
Source: G3 (Bethesda). 2016 May 16;6(7):2135–46. doi: 10.1534/g3.116.030700 (PMC4938666; doi:10.1534/g3.116.030700)

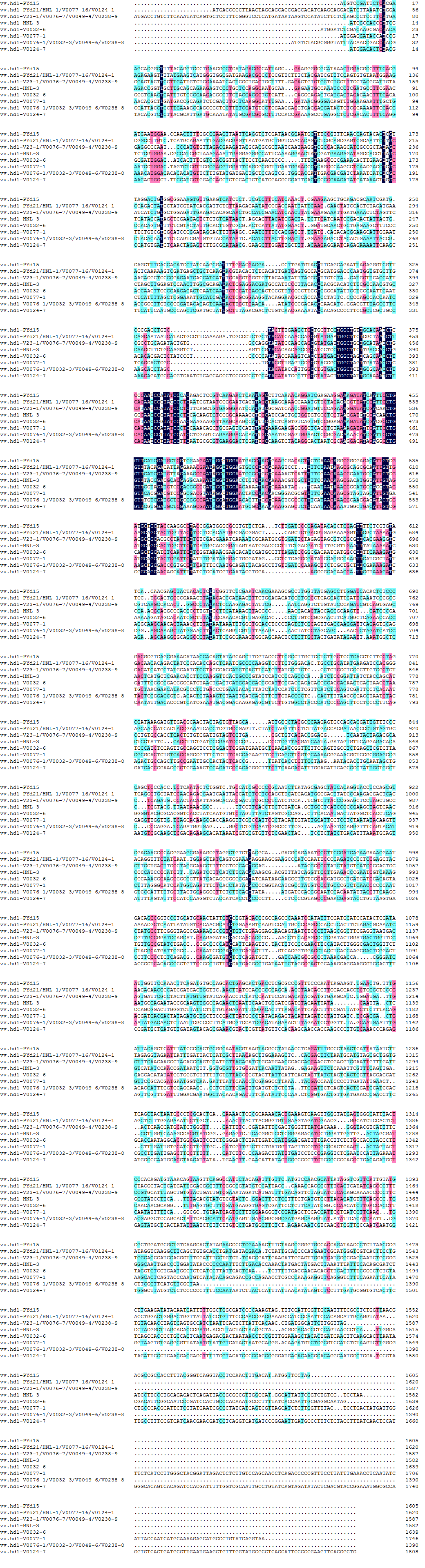

Supplement: Supplemental Material [file supp_g3.116.030700_FigureS1.tif]

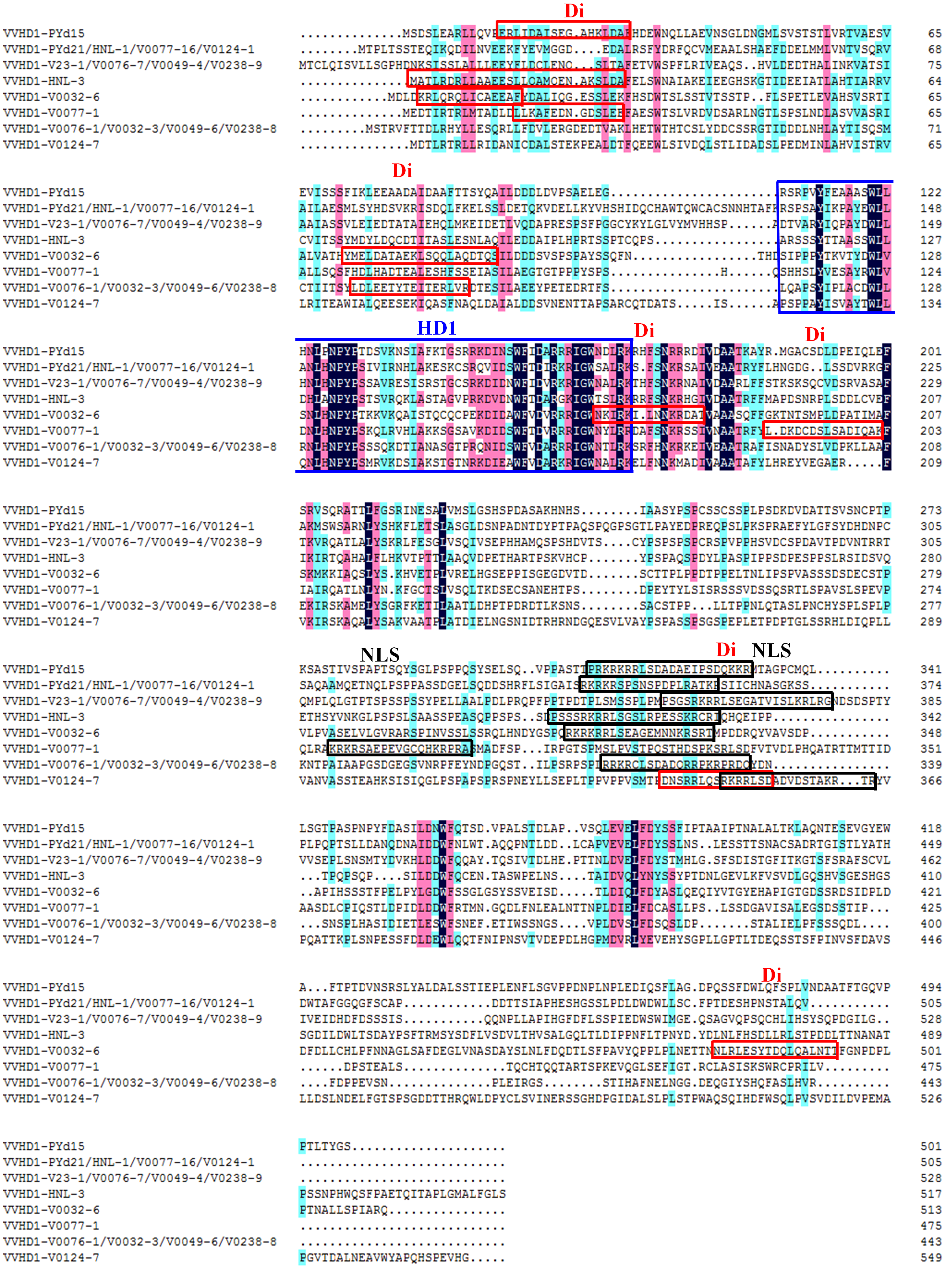

Supplement: Supplemental Material [file supp_g3.116.030700_FigureS2.tif]

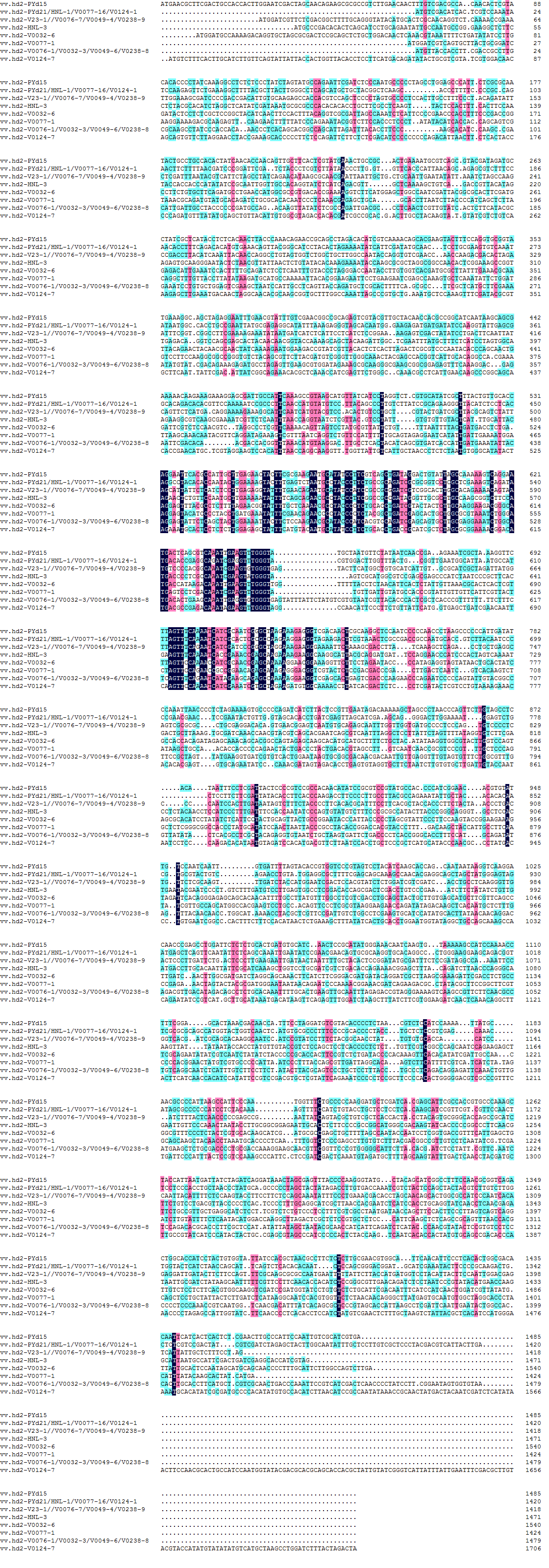

Supplement: Supplemental Material [file supp_g3.116.030700_FigureS3.tif]

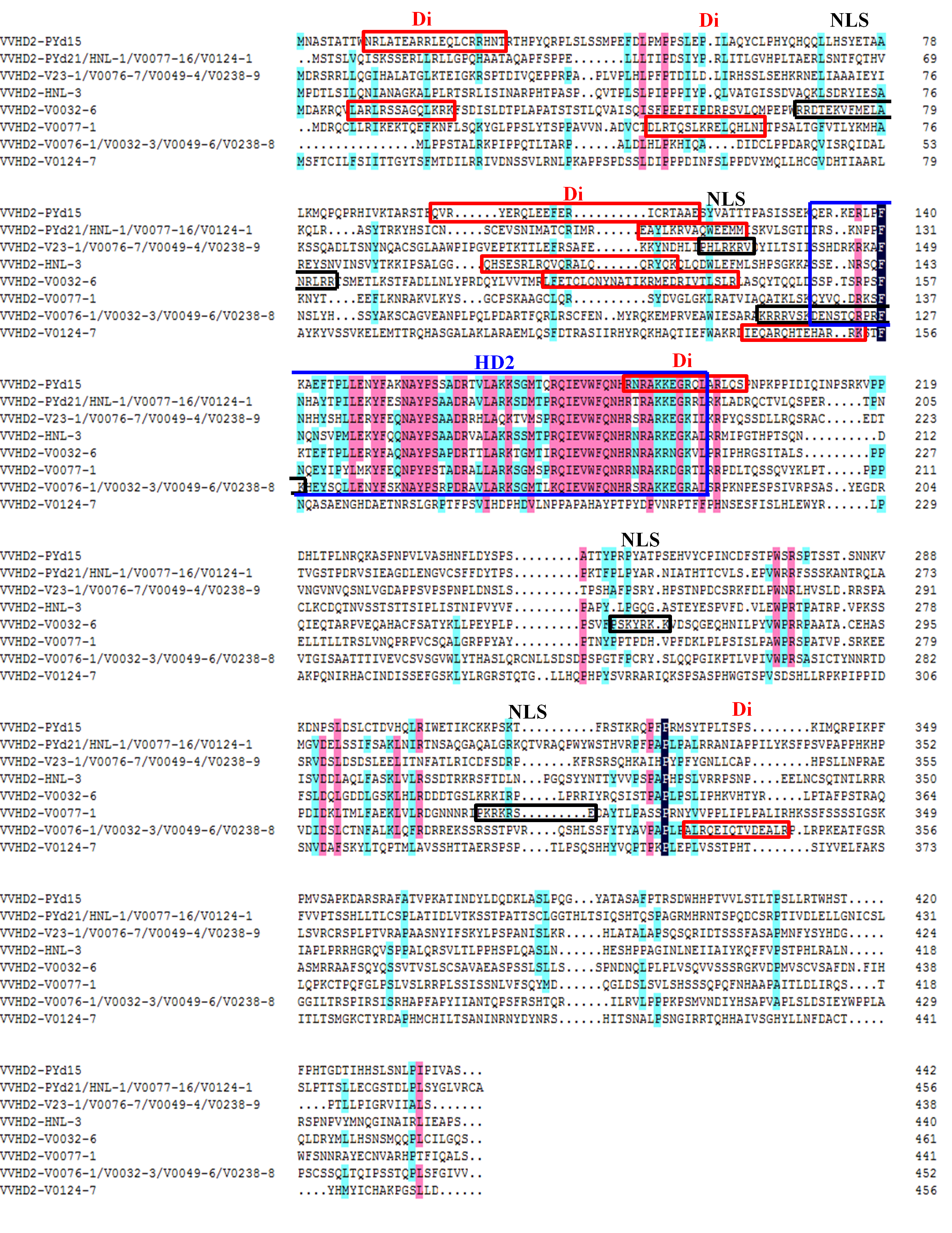

Supplement: Supplemental Material [file supp_g3.116.030700_FigureS4.tif]

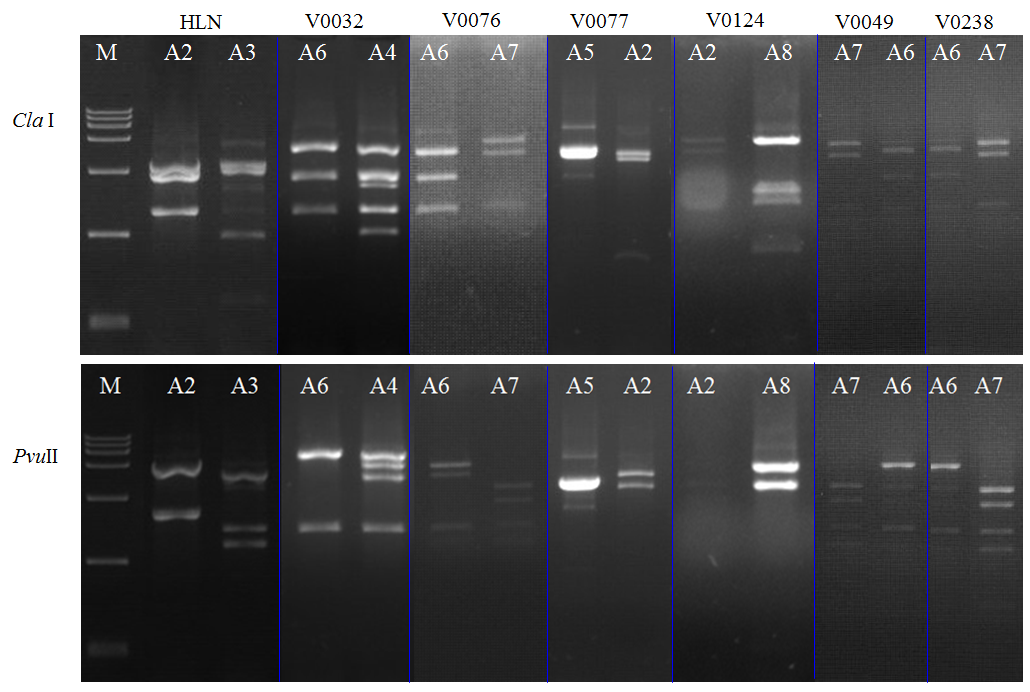

Supplement: Supplemental Material [file supp_g3.116.030700_FigureS6.tif]

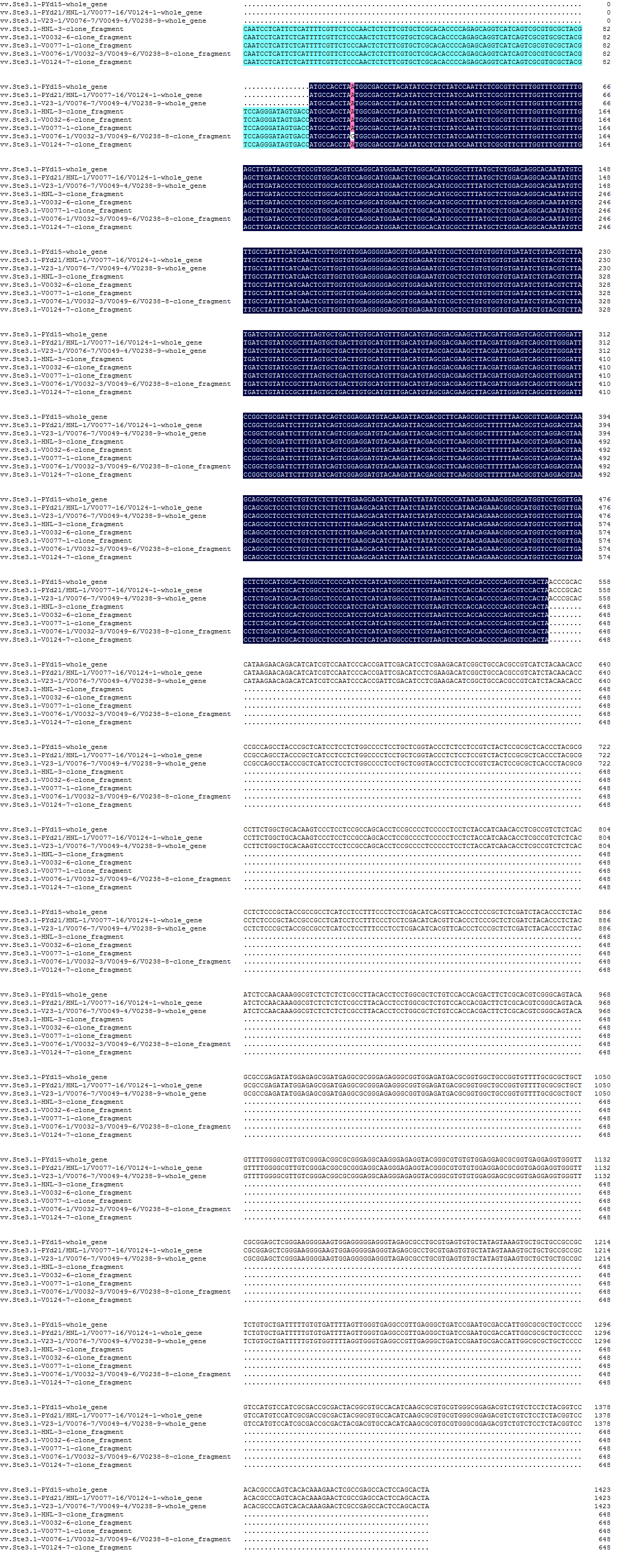

Supplement: Supplemental Material [file supp_g3.116.030700_FigureS7.tif]

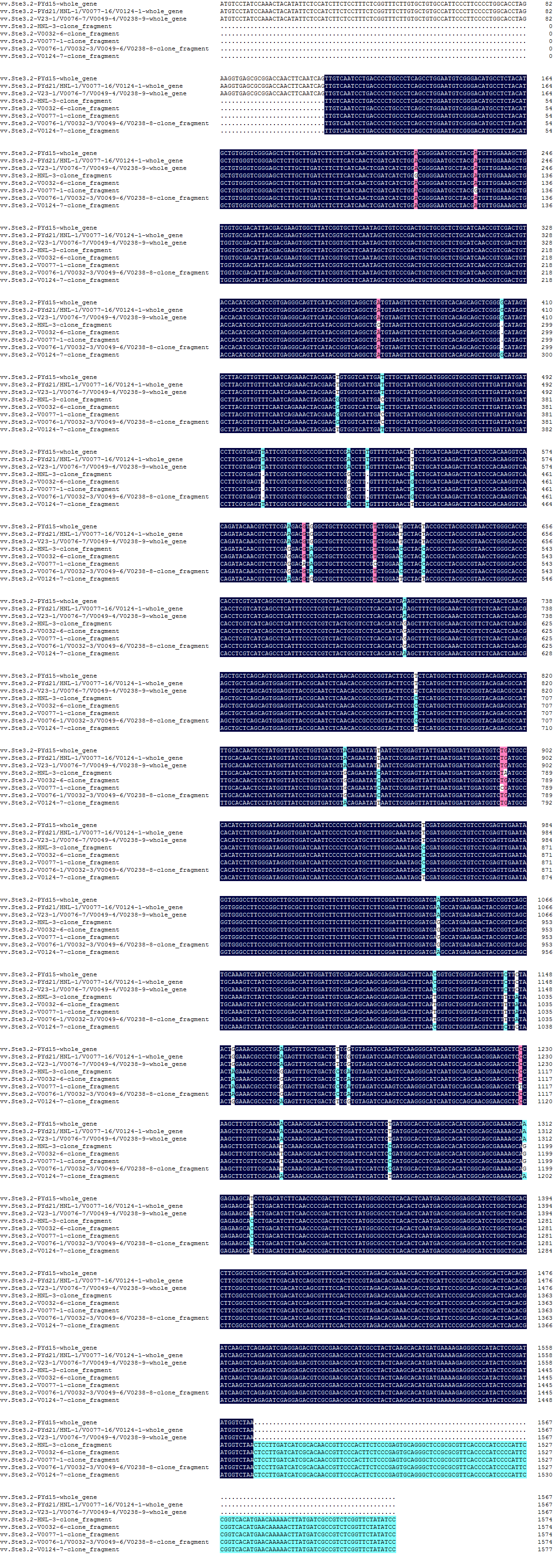

Supplement: Supplemental Material [file supp_g3.116.030700_FigureS8.tif]

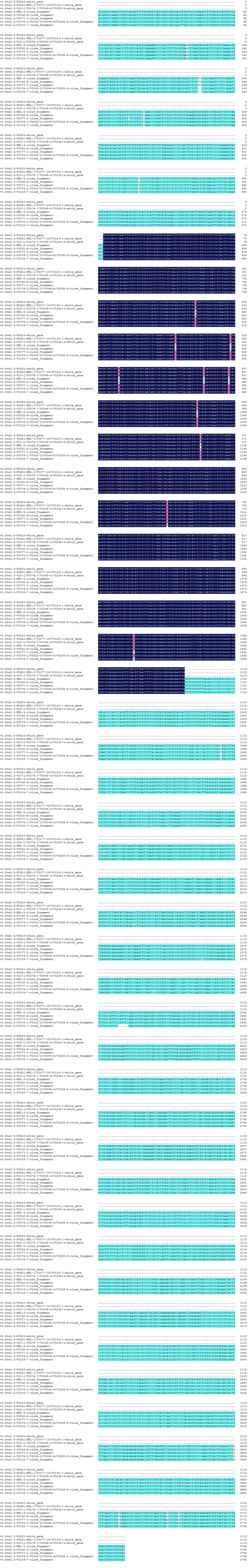

Supplement: Supplemental Material [file supp_g3.116.030700_FigureS9.tif]

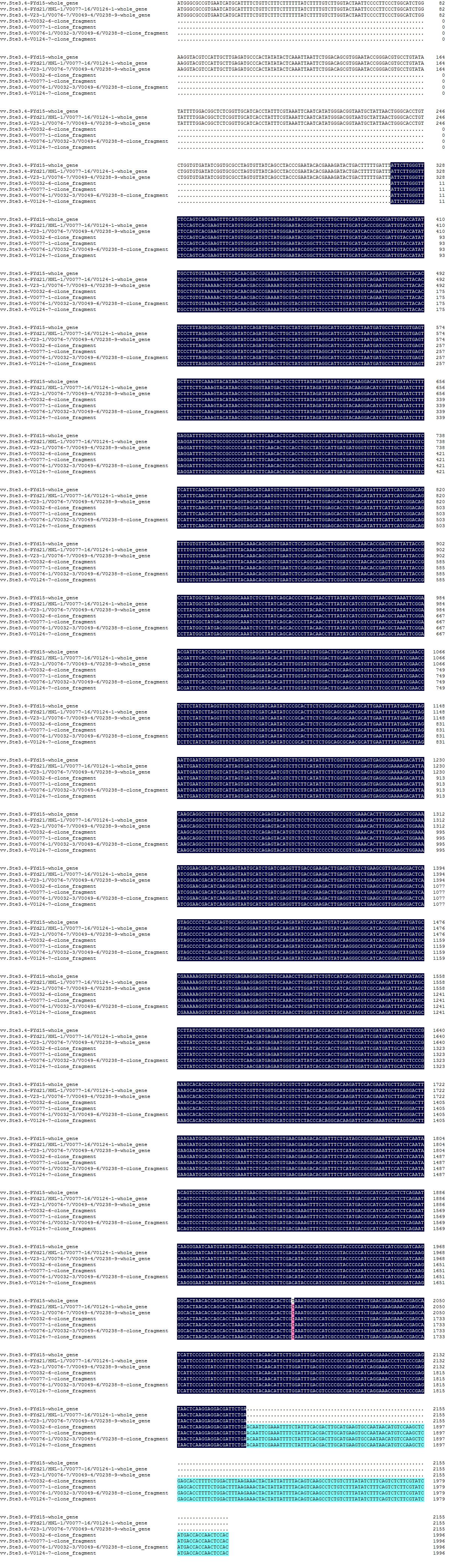

Supplement: Supplemental Material [file supp_g3.116.030700_FigureS10.tif]

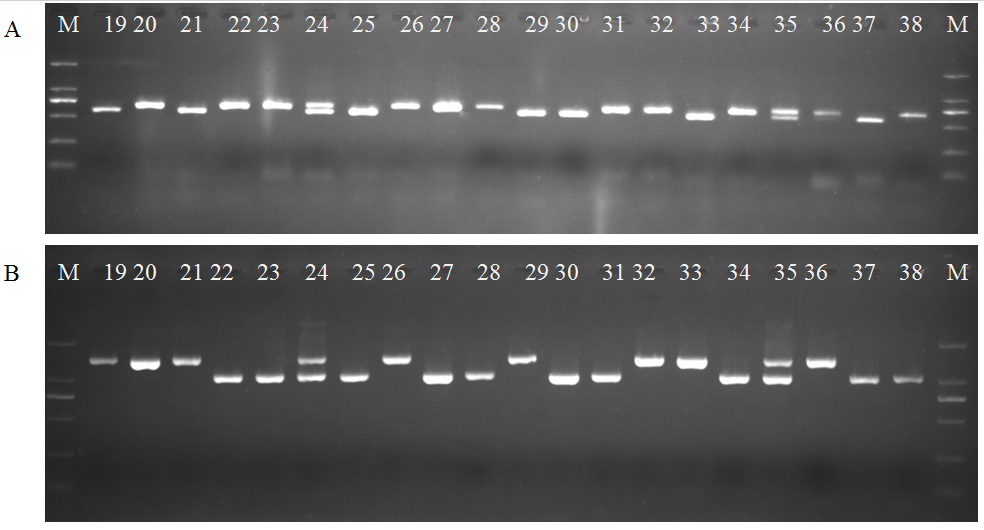

Supplement: Supplemental Material [file supp_g3.116.030700_FigureS11.tif]

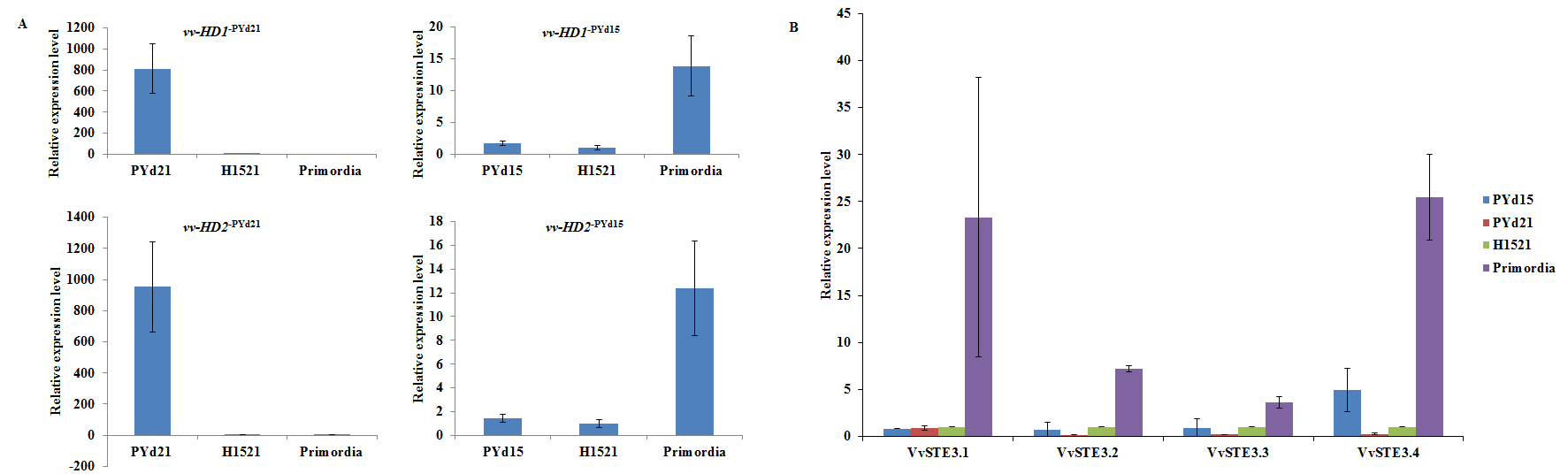

Supplement: Supplemental Material [file supp_g3.116.030700_FigureS12.tif]
